# Block copolymer synthesis in ionic liquid *via* polymerisation-induced self-assembly: a convenient route to gel electrolytes†

**DOI:** 10.1039/d3sc06717c

**Published:** 2024-02-13

**Authors:** Georgia L. Maitland, Mingyu Liu, Thomas J. Neal, James Hammerton, Yisong Han, Stephen D. Worrall, Paul D. Topham, Matthew J. Derry

**Affiliations:** a Aston Advanced Materials Research Centre, Aston University Birmingham B4 7ET UK m.derry@aston.ac.uk; b EaStCHEM School of Chemistry, The University of Edinburgh Joseph Black Building, David Brewster Road Edinburgh EH9 3FJ UK; c Department of Physics, University of Warwick Coventry CV4 7AL UK

## Abstract

We report for the first time a reversible addition–fragmentation chain transfer polymerisation-induced self-assembly (RAFT-PISA) formulation in ionic liquid (IL) that yields worm gels. A series of poly(2-hydroxyethyl methacrylate)-*b*-poly(benzyl methacrylate) (PHEMA-*b*-PBzMA) block copolymer nanoparticles were synthesised *via* RAFT dispersion polymerisation of benzyl methacrylate in the hydrophilic IL 1-ethyl-3-methyl imidazolium dicyanamide, [EMIM][DCA]. This RAFT-PISA formulation can be controlled to afford spherical, worm-like and vesicular nano-objects, with free-standing gels being obtained over a broad range of PBzMA core-forming degrees of polymerisation (DPs). High monomer conversions (≥96%) were obtained within 2 hours for all PISA syntheses as determined by ^1^H NMR spectroscopy, and good control over molar mass was confirmed by gel permeation chromatography (GPC). Nanoparticle morphologies were identified using small-angle X-ray scattering (SAXS) and transmission electron microscopy (TEM), and further detailed characterisation was conducted to monitor rheological, electrochemical and thermal characteristics of the nanoparticle dispersions to assess their potential in future electronic applications. Most importantly, this new PISA formulation in IL facilitates the *in situ* formation of worm ionogel electrolyte materials at copolymer concentrations >4% w/w *via* efficient and convenient synthesis routes without the need for organic co-solvents or post-polymerisation processing/purification. Moreover, we demonstrate that the worm ionogels developed in this work exhibit comparable electrochemical properties and thermal stability to that of the IL alone, showcasing their potential as gel electrolytes.

## Abstract 2

Block copolymer nanoparticles are synthesised via polymerisation-induced self-assembly (PISA) in ionic liquid to yield gel electrolytes, or “ionogels”, at low polymer content without the organic co-solvent or post-polymerisation processing.

## Graphical abstract

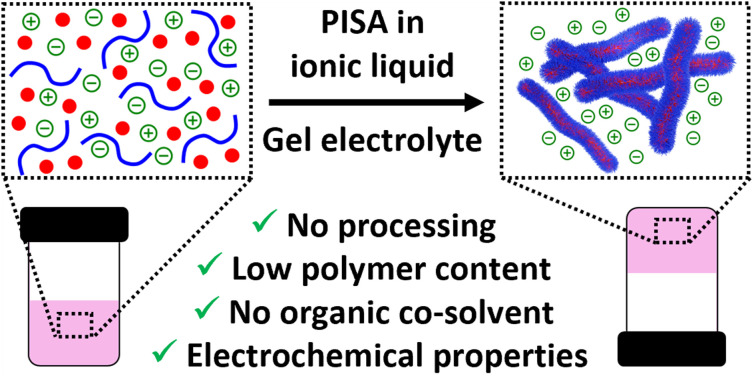

## Introduction

Ionic liquids (ILs) are typically defined as liquid electrolytes, with melting points below 100 °C, solely comprising ions.^1,2^ Compared to many traditional organic solvents, ILs exhibit advantageous properties such as high ionic conductivity, good thermal stability and low vapour pressure.^3^ Owing to the vast number of unique combinations of cations and anions, key physicochemical properties of ILs can be tailored for specific applications.^2,4^ Hence, ILs have been utilised for a diverse number of applications in electrochemistry, catalysis and analysis as well as being used as performance additives such as anti-static agents^5,6^ and dispersing agents.^4,7,8^ Due to their low vapour pressure and high thermal stability, utilising ILs as electrolyte components in batteries can potentially address flammability issues associated with traditional organic solvent-based electrolytes.^2,9^ Of particular relevance to this work, ILs have been used to develop so-called polymeric ionogels,^10,11^ which are a class of gel electrolyte formed from the immobilisation of IL in a polymer matrix. Ionogels have been used in a range of electrochemical applications such as electrolytes in lithium-ion batteries,^12–18^ dye-sensitized solar cells^19–22^ and actuators^1,23–25^ and offer an alternative to liquid electrolytes thus significantly reducing the risk of potentially hazardous leakage.

There have been several different approaches reported that use polymers to prepare ionogels. Generally, physically crosslinked or chemically crosslinked ionogels can be generated depending on the preparation method.^26,27^ For example, chemically crosslinked ionogels can be prepared by (i) the polymerisation of vinyl monomers in an IL with the addition of a crosslinker to immobilise the IL^16,28–32^ or (ii) utilising poly(ionic liquids).^33–36^ In contrast, physically crosslinked ionogels have been prepared utilising (i) hydrogen bonding,^37–40^ (ii) ion–dipole interactions^39^ or (iii) the self-assembly of block copolymers^9–11,41–43^ or random copolymers^44^ in an IL with the aid of a co-solvent.

Over the past few decades, reversible addition–fragmentation chain transfer (RAFT) polymerisation has emerged as a popular method of reversible deactivation radical polymerisation (RDRP) to generate an extensive range of functional, well-defined diblock copolymers.^45^ Exploiting its versatility, RAFT-mediated polymerisation-induced self-assembly (PISA)^46–49^ has been demonstrated as a convenient tool to synthesise block copolymer nanoparticles in a range of media such as alcohols,^50–52^ non-polar solvents^53–55^ and water.^56–61^ PISA has many advantages over traditional block copolymer self-assembly methods, for example it can be conducted at high copolymer concentrations (up to 50% w/w)^62,63^ and does not require post-polymerisation processing steps such as solvent/pH switching and film rehydration.^56,64^ In a typical PISA reaction, a soluble macromolecular chain transfer agent (macro-CTA, *i.e.* the stabilizer block) is chain extended using a monomer that polymerises to form an insoluble block, resulting in the formation of nanoparticles comprising amphiphilic AB diblock copolymer chains.^65^ PISA can be conducted under dispersion or emulsion conditions, depending on whether the monomer that is polymerised to form the insoluble structure-directing block is miscible (dispersion)^46,54,65–67^ or immiscible (emulsion).^68,69^

To date, there have been very few reports of PISA being conducted in IL. Zhang and Zhu^3^ reported the synthesis of a series of diblock copolymers *via* RAFT dispersion polymerisation in a relatively hydrophobic IL, 1-butyl-3-methylimidazolium hexafluorophosphate, [BMIM][PF_6_], whereby a functionalised poly(ethylene glycol) macro-CTA was chain extended using three different monomers (2-hydroxyethyl methacrylate, styrene or *n*-butyl methacrylate). This seminal work demonstrated the potential for preparing dispersions of functional block copolymer nanoparticles in IL *via* convenient PISA protocols, however only isotropic nanoparticles (spheres or vesicles) were successfully obtained as confirmed by transmission electron microscopy (TEM) studies. We do note one instance in this study where the formation of so-called ‘rod-like aggregates’ or ‘stretched vesicles’ species was demonstrated, however these anisotropic objects were only present as a minor population. Similarly, Zhou *et al.*^70^ reported the preparation of poly(ethylene glycol)-*b*-polystyrene (PEG-*b*-PS) nanospheres and vesicles *via* RAFT dispersion polymerisation in [BMIM][PF_6_]. In this further study, the same PEG-*b*-PS block copolymer was also synthesised *via* alcoholic PISA in order to assess the effect of solvent choice on polymerisation kinetics. It was demonstrated that block copolymer synthesis conducted in this IL was faster: 95% monomer conversion was achieved within 12 hours in [BMIM][PF_6_] compared to only 20% and 65% within the same time frame for reactions conducted in methanol and a methanol/water mixture, respectively. Demarteau *et al.*^71^ reported the synthesis of poly(ionic liquid)-containing block copolymers *via* aqueous emulsion PISA, yielding spherical particles with diameters >300 nm as confirmed by TEM analysis. Specifically, the hydrophilic IL diallyldimethylammonium chloride (DADMAC) was functionalised to form mono- and di-functional poly(diallyldimethylammonium chloride) (PDADMAC) macro-CTAs that were subsequently used to prepare di- and triblock copolymers *via* RAFT aqueous emulsion polymerisation of styrene to yield spherical polyelectrolyte latex particles. Ionogel membranes were prepared in this previous study *via* post-polymerisation formulation, where the particles dispersed in aqueous medium were mixed with ionic liquid electrolyte with the aid of toluene co-solvent for 10 hours before being cast onto Teflon.

Despite PISA formulations being developed in ILs, there have been no reports of a system that yields worm-like micelles, which typically occupy phase space where the degree of polymerisation of the structure-directing block lies between those for spherical micelles and vesicles.^62^ Worm-like micelles provide a route to soft gel materials at lower copolymer concentrations (≥3% w/w)^72^ than the polymer concentrations typically required for ionogel preparation (up to 10% w/w).^11^ Whilst there have been recent advances in this area, developing PISA formulations that provide access to worm gels in ILs would represent a much more facile route to functional ionogel materials using straightforward synthesis and preparation routes without the need for post-polymerisation processing or purification.

**Scheme 1:**
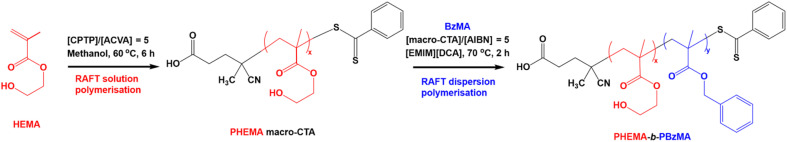
Synthesis of poly(2-hydroxymethacrylate) (PHEMA) macro-CTA *via* RAFT solution polymerisation in methanol at 60 °C, followed by RAFT dispersion polymerisation of benzyl methacrylate (BzMA) in 1-ethyl-3-methylimidazolium dicyanamide ([EMIM][DCA]) at 70 °C to yield PHEMA-*b*-PBzMA diblock copolymers.

Herein, we report for the first time a RAFT-PISA formulation that yields diblock copolymer spheres, worms and vesicles in IL. Specifically, poly(2-hydroxyethyl methacrylate)-*b*-poly(benzyl methacrylate) (PHEMA-*b*-PBzMA) block copolymer nanoparticles were synthesised *via* RAFT dispersion polymerisation in 1-ethyl-3-methylimidazolium dicyanamide, [EMIM][DCA] (Scheme 1). Detailed characterisation was conducted, including ^1^H nuclear magnetic resonance (NMR) spectroscopy, gel permeation chromatography (GPC), dynamic light scattering (DLS), transmission electron microscopy (TEM), small-angle X-ray scattering (SAXS), oscillatory rheology, thermogravimetric analysis (TGA) and electrochemical impedance spectroscopy (EIS). For the first time, this new PISA formulation facilitates the *in situ* formation of self-standing worm ionogel electrolyte materials at copolymer concentrations >4% w/w *via* efficient and convenient synthesis routes without the need for organic co-solvents, crosslinkers, post-polymerisation processing or purification. Importantly, we demonstrate that the worm ionogels developed in this work exhibit comparable electrochemical properties to that of the ionic liquid alone, showcasing their potential as gel electrolytes.

## Experimental

### Materials

2-Hydroxyethyl methacrylate (HEMA) and benzyl methacrylate (BzMA) were purchased from Sigma Aldrich and passed through a basic alumina column prior to use in order to remove the inhibitor. 2,2′-Azobisisobutyronitrile (AIBN) was purchased from Molekula and was recrystallised from methanol prior to use. 4-Cyano-4-(phenylcarbonothioylthio) pentanoic acid (CPTP) RAFT agent was purchased from Sigma Aldrich and used as received. Reagent grade methanol and diethyl ether were purchased from Fisher Scientific. Dimethyl sulfoxide-d_6_ and methanol-d_4_ for ^1^H NMR analysis were purchased from Goss Scientific. 1-Ethyl-3-methylimidazolium dicyanamide [EMIM][DCA] was acquired from BASF.

### Synthesis of poly(2-hydroxyethyl methacrylate) macromolecular chain transfer agent *via* RAFT solution polymerisation

The synthesis of the PHEMA_30_ macro-CTA at 50% w/w solids was conducted as follows. A 100 mL round-bottomed flask was charged with 2-hydroxyethyl methacrylate (HEMA; 20 g; 154 mmol), 4-cyano-4-(phenylcarbonothioylthio)pentanoic acid (CPTP; 0.859 g; 3.0 mmol), 2,2′-azobisisobutyronitrile (AIBN; 100.9 mg; 615 μmol; CPTP/AIBN molar ratio = 5) and methanol (21 g). The sealed reaction flask was purged with nitrogen for 30 minutes prior to being placed in a preheated oil bath at 60 °C and stirred for 6 hours. The resulting PHEMA (HEMA conversion = 40%; *M*_n_ = 8000 g mol^−1^, *Đ*_M_ = *M*_w_/*M*_n_ = 1.25) was purified by twice precipitating into a ten-fold excess of diethyl ether and dried on a rotary evaporator until all solvent was removed as judged by ^1^H NMR spectroscopy (see Fig. S2†). The resulting PHEMA macro-CTA was obtained as a pink solid. The mean degree of polymerisation (DP) of this macro-CTA was calculated to be 30 using ^1^H NMR spectroscopy by comparing the integrated signals corresponding to the five CPTP aromatic protons at 7.2–8.0 ppm relative to the peak at 4.0–4.1 ppm corresponding to the two oxymethylene protons of PHEMA (see Fig. S2†).

### Synthesis of poly(2-hydroxyethyl methacrylate)-*block*-poly(benzyl methacrylate) (PHEMA-*b*-PBzMA) diblock copolymer *via* RAFT dispersion polymerisation in 1-ethyl-3-methylimidazolium dicyanamide

A typical RAFT dispersion polymerisation for the synthesis of PHEMA_30_–PBzMA_291_ at 15% w/w solids was conducted as follows. Benzyl methacrylate (BzMA; 0.52 g; 2.96 mmol), 2,2′-azobisisobutyronitrile (AIBN; 0.3 mg; 1.97 μmol), PHEMA_30_ macro-CTA (0.04 g; 9.87 μmol; macro-CTA/initiator molar ratio = 5; PBzMA target DP = 300) and 1-ethyl-3-methylimidazolium dicyanamide ([EMIM][DCA]; 3.18 g) were added to a 14 mL sample vial. The sealed reaction mixture was purged with nitrogen for 30 minutes prior to being placed in a preheated oil bath at 70 °C whilst stirring for 2 hours (BzMA conversion = 98%; *M*_n_ = 52 900 g mol^−1^, *Đ*_M_ = 1.35).

### ^1^H NMR spectroscopy

^1^H NMR spectra were obtained in either MeOD-d_4_ or DMSO-d_6_ using a Bruker Avance Neo 300 MHz spectrometer. Typically 16 scans were averaged per spectrum and all chemical shifts are expressed in ppm.

### Gel permeation chromatography

Molecular weight distributions were obtained by using an Agilent Infinity II multi-detector gel permeation chromatography (GPC) instrument comprising a guard column and two PL gel mixed-C columns. The mobile phase contained 0.10% w/v LiBr in HPLC grade DMF and the flow rate was fixed at 1 mL min^−1^ at 80 °C. The GPC was calibrated using near-monodispersed poly(methyl methacrylate) standards (*M*_p_ range = 535–1 591 000 g mol^−1^).

### Dynamic light scattering

Dynamic light scattering (DLS) studies were conducted using a Zetasizer Nano ZS instrument (Malvern Panalytical, UK) at a fixed scattering angle of 173°. The block copolymer dispersions were diluted in [EMIM][DCA] (refractive index = 1.51 as determined by Soriano *et al.*,^73^ viscosity = 14.6 cP) to 0.10% w/w prior to light scattering studies at 25 °C. The polydispersity index (PDI) and average diameter (*D*) were calculated, and data were averaged over three sets of approximately thirteen runs each of 30 seconds duration.

### Transmission electron microscopy

Bright field transmission electron microscopy (TEM) studies were conducted using a JEOL2100 instrument operating at 200 kV. Prior to analysis, block copolymer dispersions were diluted with [EMIM][DCA] to 0.15% w/w, placed on carbon-coated copper grids, blotted using filter paper and allowed to dry overnight, following a previously reported protocol.^74^ No staining agent was required.

### Oscillatory rheology

The loss and storage moduli were measured as a function of shear strain between 0.1% and 100% at a fixed angular frequency of 6.28 rad s^−1^ to assess the gel strength. The moduli were also measured as a function of frequency between 1 rad s^−1^ and 100 rad s^−1^ at a fixed complex shear strain of 1.0%. All measurements were conducted at 25 °C.

### Small-angle X-ray scattering

Small-angle X-ray scattering (SAXS) patterns were recorded for 1.0% w/w copolymer dispersions in [EMIM][DCA] in 1.5 mm diameter polycarbonate capillaries at a synchrotron source (beamline B21,^75^ Diamond Light Source, UK) using monochromatic X-ray radiation (X-ray wavelength *λ* = 0.9408 Å, sample-to-detector distance of 3.712 m corresponding to scattering vector *q* ranging from 0.0045 to 0.34 Å^−1^) and an EigerX 4M detector (Dectris, Switzerland). Scattering data were reduced using standard protocols from the beamline and were further analyzed using Irena SAS macros for Igor Pro.^76^ Background-subtracted SAXS data were fitted to an appropriate model (or a combination models when a mixed phase is observed): (i) Gaussian chains,^77^ (ii) spherical micelles,^78^ (iii) worm-like micelles,^78^ (iv) vesicles^79^ (see ESI† for detailed information of models and fitting summaries).

### Helium pycnometry

The solid-state density of PHEMA_30_ macro-CTA was determined using a Micromeritics AccuPyc II 1345 pycnometer at 20 °C using a 1 cm^3^ cup. The instrument was calibrated using a 0.718537 cm^3^ ball bearing calibrant. The reported value was an average of 10 measurements. The solid-state density of PBzMA was previously determined using helium pycnometry to be 1.15 g cm^−3^.^80^

### Electrochemical impedance spectroscopy

Electrochemical impedance spectroscopy (EIS)^81^ measurements were performed in a symmetrical two electrode configuration. Stainless steel spacers (15.5 mm diameter, 0.5 mm thickness) were used as the electrodes and were separated by either Whatman Grade 1 Qualitative Filter Paper soaked in [EMIM][DCA] or an ionogel sample, within a sealed CR2032 coin cell. EIS was performed using a PGSTAT302N potentiostat (Metrohm Autolab B.V., The Netherlands) over the frequency range 1 MHz to 200 mHz with a 10 mV RMS perturbation voltage. Bulk resistance values were extracted from Nyquist plots by taking the high frequency intercept of the *x*-axis. Measurements were performed in triplicate.

### Thermogravimetric analysis

Thermogravimetric analysis (TGA) was used to assess the thermal behaviour of the gels, focusing on their thermal degradation temperature relative to the IL alone. The thermal degradation was measured by monitoring the relative change in mass as a function of increasing temperature. TGA was performed using a PerkinElmer TGA 8000 under nitrogen atmosphere (flow rate 40 mL min^−1^). All samples were heated from 150 °C to 600 °C at a rate of 10 °C min^−1^.

## Results and discussion

### Synthesis of PHEMA macromolecular chain transfer agent

A PHEMA macromolecular chain transfer agent (macro-CTA) with a mean degree of polymerisation (DP) of 30 was synthesised *via* RAFT solution polymerisation in methanol at 60 °C using 4-cyano-4-(phenylcarbonothioylthio)pentanoic acid (CPTP) as a chain transfer agent (Scheme 1). Commonly in macro-CTA syntheses, polymerisations are quenched before reaching high (≥80%) monomer conversion in order to retain RAFT end groups by avoiding monomer-starved conditions,^82^ thus enabling high blocking efficiencies in subsequent block copolymer syntheses. In this study, the RAFT solution polymerisation of HEMA was quenched after 6 hours, affording a monomer conversion of 40% as judged by ^1^H NMR spectroscopy (Fig. S1†). This yielded a relatively short PHEMA_30_ macro-CTA stabiliser block, which is often favourable for the proceeding PISA syntheses when targeting higher order nanoparticle morphologies (*i.e.* worms and vesicles).^83^ The retention of RAFT end groups was confirmed by the ^1^H NMR spectrum of the purified macro-CTA by presence of the aromatic protons within the Z-group of the RAFT agent (see Fig. S2†). The synthesised PHEMA_30_ macro-CTA exhibited a relatively narrow molecular weight distribution (*Đ*_M_ = 1.25), indicating that this RAFT solution polymerisation of HEMA was well-controlled. Additionally, helium pycnometry measurements indicated a solid-state PHEMA_30_ density of 1.26 g cm^−3^, which is in good agreement with previously reported values.^84^ Prior to chain extending the PHEMA_30_ macro-CTA, the solubility of the macro-CTA was assessed in [EMIM][DCA]. The potential for the PHEMA_30_ macro-CTA to be an appropriate stabiliser block for PISA syntheses in this hydrophilic ionic liquid was confirmed based on its good solubility at 10% w/w.

### Synthesis and characterisation of PHEMA-*b*-PBzMA block copolymers in [EMIM][DCA]

**Fig. 1:**
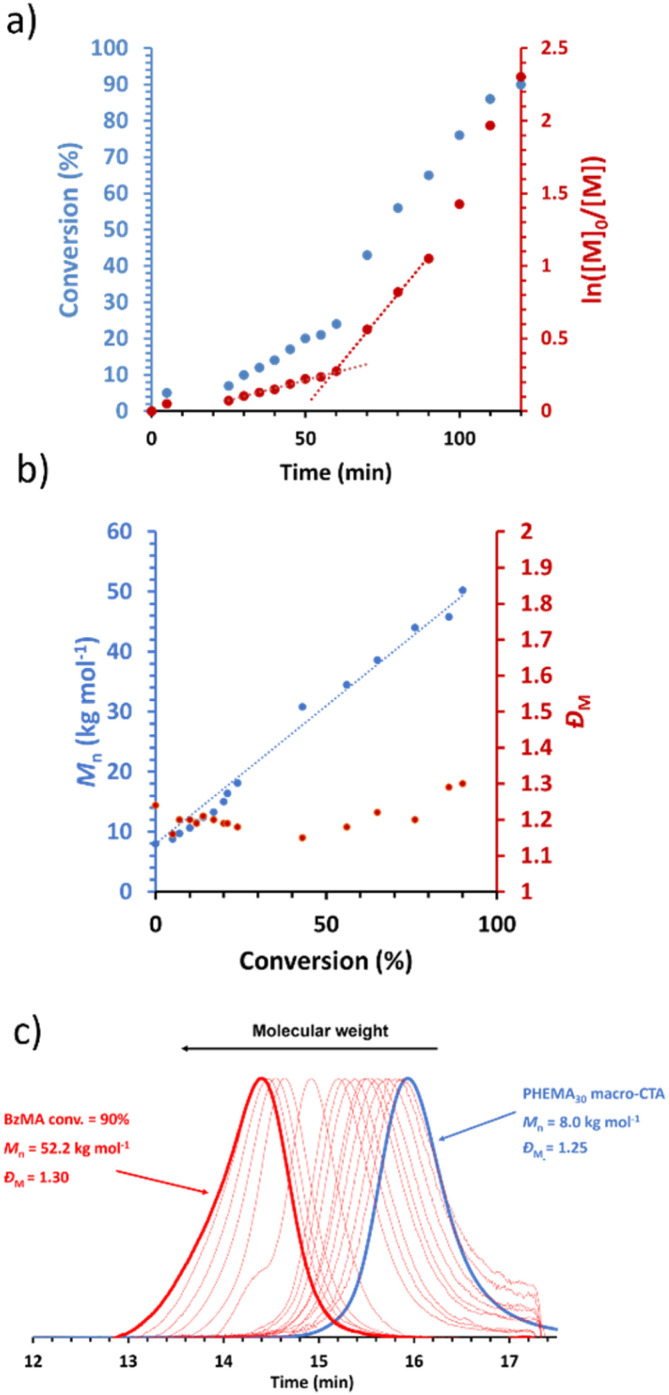
Kinetic study for the RAFT dispersion polymerisation of BzMA in [EMIM][DCA] at 15% w/w solids using a PHEMA_30_ macro-CTA: (a) BzMA conversion *vs.* time (blue data) and semi-log kinetic (red data) plots; (b) *M*_n_ and *Đ*_M_*vs.* BzMA conversion. Dashed blue line indicates linear progression of molar mass growth; (c) DMF GPC chromatograms. GPC data was obtained against poly(methyl methacrylate) standards.

A representative kinetic study of the chain extension of PHEMA_30_ with benzyl methacrylate (BzMA) in [EMIM][DCA] at 70 °C and 15% w/w solids was conducted when targeting a PBzMA DP of 300 (Fig. 1). Aliquots from the reaction solution were taken at 5 minute intervals during the first hour, then every 10 minutes thereafter to monitor the polymerisation kinetics. An induction period up to 25 minutes is observed, followed by a relatively slow rate of polymerisation up to 60 minutes, as is common for RAFT solution polymerisation (*i.e.* where block copolymer chains are fully soluble). After 60 minutes, a significant rate enhancement was observed, which typically indicates the onset of micellar nucleation where a critical length of the solvophobic block is reached, above which the propagating block becomes insoluble. This rate enhancement arises due to the preferential migration of unreacted monomer into the nanoparticle cores within which the polymerisation proceeds, thus providing a higher effective local monomer concentration at the site of polymerisation.^62,85^ In the present PISA formulation, the critical PBzMA DP for the assembly of PHEMA_30_-*b*-PBzMA_*y*_ nanoparticles during PISA in [EMIM][DCA] was estimated to be 72. At this critical point in the PISA synthesis, the observed rate of polymerisation increased by a factor of approximately 6 (Fig. 1a). GPC data obtained showed good control over the polymerisation as judged by the low mass dispersities throughout and linear evolution of molecular weight with monomer conversion (Fig. 1b). Additionally, GPC traces confirm efficient chain extension of the PHEMA_30_ macro-CTA during the RAFT dispersion polymerisation of benzyl methacrylate in [EMIM][DCA] (Fig. 1c).

**Fig. 2:**
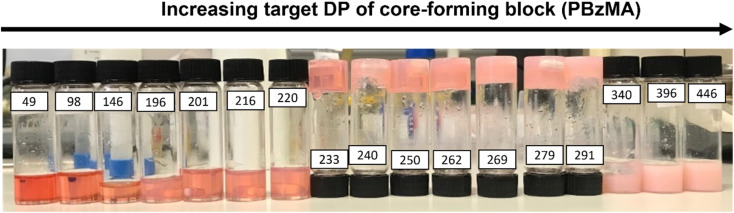
Digital image showing the physical appearance of the series of PHEMA_30_-*b*-PBzMA_*y*_ block copolymer dispersions in [EMIM][DCA] at 15% w/w solids. Number labels on sample vials denote the actual PBzMA core-forming block DP, as determined using ^1^H NMR spectroscopy. Inverted sample vials indicate free-standing gels.

Following this kinetic study, the optimum polymerisation time for complete monomer conversion was identified as 2 hours. Thus, a series of PHEMA_30_-*b*-PBzMA_*y*_ block copolymers at 15% w/w with varying target DPs of the core-forming PBzMA block (*i.e.* ‘*y*’ values) was synthesised (Table S1† and Fig. 2). Importantly, high monomer conversions (≥96%) were obtained for all syntheses as determined by ^1^H NMR spectroscopy. A range of dispersions were formed (Fig. 2), with initial observations indicating that varying morphologies were synthesised based on comparisons to previous PISA formulations.^80^ Transparent fluids formed at lower PBzMA DPs typically indicate the presence of either dissolved polymer chains (*i.e.* no PISA occurred) or spheres. Dispersions of PHEMA_30_-*b*-PBzMA_*y*_ where *y* = 233–317 yielded free-standing gels, suggesting the formation of worm-like nanoparticles that can form an extended percolating gel network.^72^ Beyond this gel phase, turbid free-flowing solutions at higher PBzMA DPs (>330) are characteristic of the presence of vesicles.

**Fig. 3:**
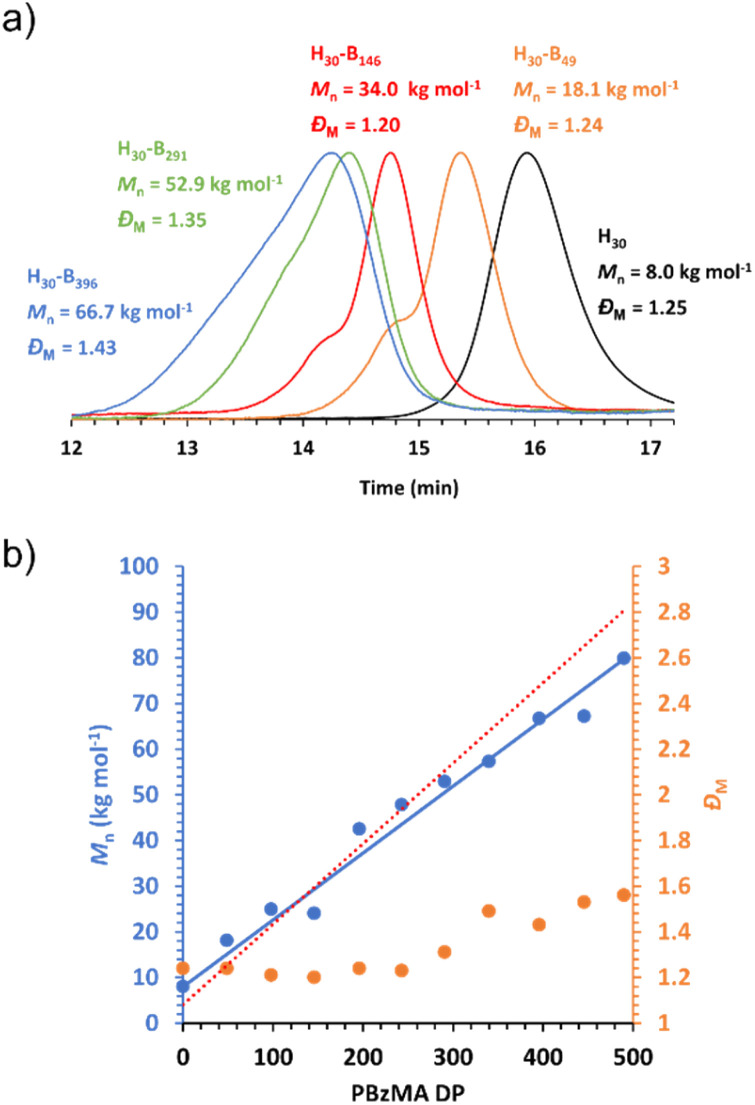
DMF GPC data obtained for PHEMA_30_-*b*-PBzMA_*y*_ block copolymers synthesised *via* RAFT dispersion polymerisation of benzyl methacrylate in [EMIM][DCA] at 15% w/w solids. GPC data was obtained against poly(methyl methacrylate) standards. (a) Chromatograms obtained for a selection of PHEMA_30_-*b*-PBzMA_*y*_ block copolymers, where PHEMA is denoted as H and PBzMA is denoted as B. (b) *M*_n_*vs.* PBzMA DP (blue), where the blue line indicates the line of best fit, and *Đ*_M_*vs.* PBzMA DP (orange), where the red dashed line indicates the theoretical *M*_n_*vs.* PBzMA DP. Theoretical *M*_n_ and DP were obtained by ^1^H NMR spectroscopy, and actual *M*_n_ and *Đ*_M_ were obtained by GPC analysis.

As expected, the molecular weight of the block copolymer increases as the DP of the PBzMA core-forming block increases. GPC confirms a clear shift to shorter retention times for the block copolymers compared to the PHEMA_30_ macro-CTA with negligible precursor macro-CTA chains remaining in all block copolymer samples, characteristic of both an increase in molecular weight and efficient macro-CTA chain-extension during the PISA synthesis (Fig. 3a). The slight discrepancies between the molecular weight obtained by GPC and that obtained *via*^1^H NMR spectroscopy (Fig. 3b) can be attributed to the poly(methyl methacrylate) calibration standards used for GPC analysis.^66^ In contrast to the molar mass dispersity of the PHEMA_30_ macro-CTA (*Đ*_M_ = 1.25), the dispersities obtained for the block copolymers shown in Fig. 3 were similar up to a target PBzMA DP of 200 (*Đ*_M_ ≤ 1.24), indicating good control of the molecular weight distribution during the PISA syntheses. Some loss of control was observed when targeting higher PBzMA DPs, for example a target DP of 400 where *Đ*_M_ = 1.43, which is often a characteristic of block copolymers synthesised *via* RAFT-PISA when targeting relatively high core-forming block DPs.^65,68,72^ The high molecular weight tailing observed in GPC traces when targeting higher PBzMA DPs is most likely due to some low level of termination by combination as has been previously observed for PISA formulations involving BzMA.^86^

### Characterisation of PHEMA_30_-*b*-PBzMA_*y*_ nanoparticles

**Fig. 4:**
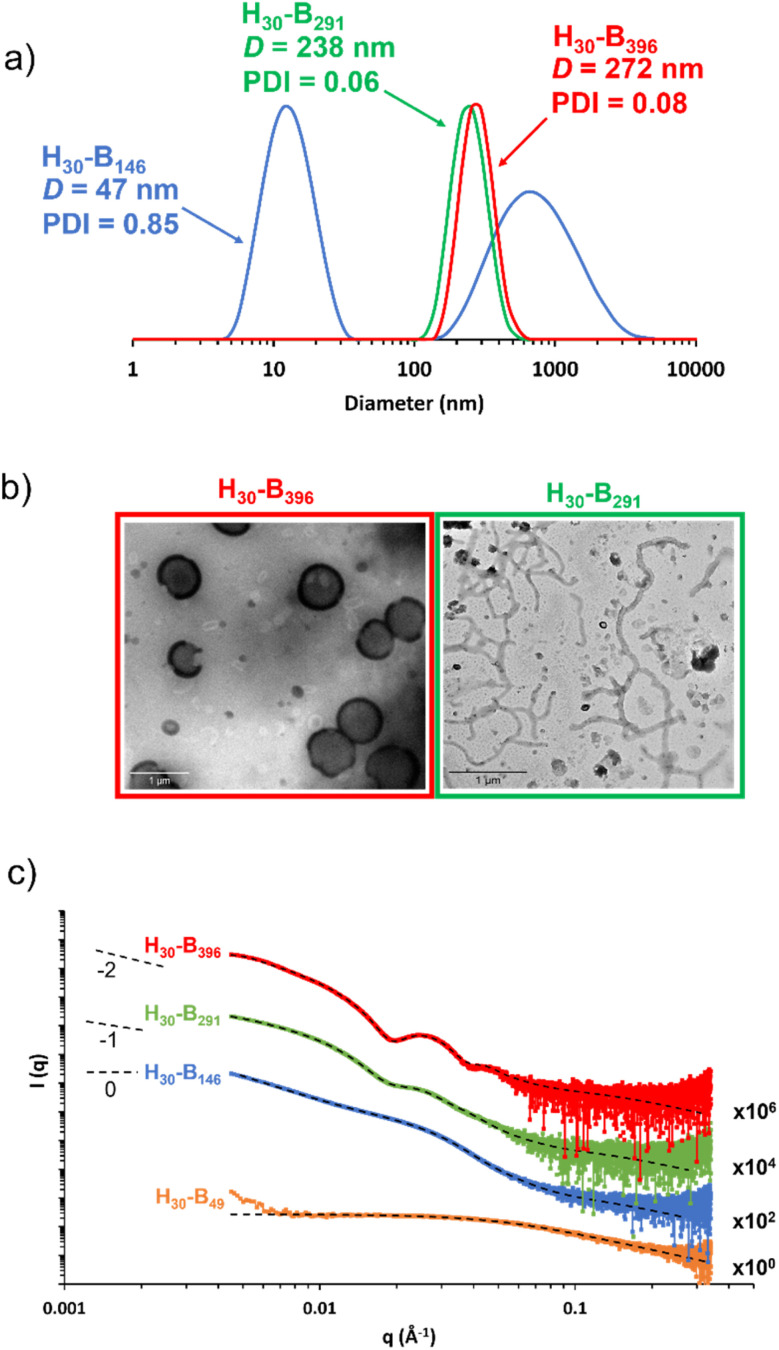
(a) DLS data obtained for 0.15% w/w dispersions of PHEMA_30_-*b*-PBzMA_396_ (red), PHEMA_30_-*b*-PBzMA_291_ (green) and PHEMA_30_-*b*-PBzMA_146_ (blue) in [EMIM][DCA], (b) TEM images obtained for 0.15% w/w dispersions of PHEMA_30_-*b*-PBzMA_396_ (red) and PHEMA_30_-*b*-PBzMA_291_ (green) in [EMIM][DCA], and (c) background-subtracted SAXS patterns recorded at 1.0% w/w for PHEMA_30_-*b*-PBzMA_*y*_ chains (orange), spheres and chains mixture (blue), spheres and worms mixture (green) and vesicles (red) in [EMIM][DCA]. Dashed lines represent model fits obtained, where a combination of the spherical micelle, worm-like micelle and vesicle models were employed. Gradients of 0, −1 and −2 are shown as a guide to the eye indicate the presence of spheres, worms and vesicles, respectively.

DLS studies were conducted to characterise the PHEMA_30_-*b*-PBzMA_*y*_ nanoparticles in [EMIM][DCA], indicating the formation of nano-objects ranging in DLS diameter from ∼40 nm to ∼700 nm (see Table S1 and Fig. S4†). Whilst DLS analysis is not ideally suited to the analysis of anisotropic nanoparticles, the apparent diameter returned can provide a ‘sphere equivalent’ size. It is noteworthy that nanoparticles formed when targeting PBzMA DPs of 340–450 were ∼250–350 nm in diameter with narrow size distributions (DLS polydispersity index, PDI ≤ 0.08) which, given that this PBzMA DP range is above the range where gels are obtained, suggests the formation of vesicles. DLS size distributions of selected PHEMA_30_-*b*-PBzMA_*y*_ dispersions are shown in Fig. 4a.

The presence of nanoparticles was also confirmed by TEM and SAXS (Fig. 4b and c). In particular, TEM analysis of PHEMA_30_-*b*-PBzMA_396_ in [EMIM][DCA] indicated the presence of vesicles, which was further evidenced by SAXS analysis by fitting background-subtracted data to a well-established vesicle model.^79^ For this sample, SAXS data were successfully fitted using a vesicle model alone indicating a pure phase of vesicles with a mean overall vesicle diameter of 315 nm, which is comparable to the overall DLS diameter observed by DLS, and the mean membrane thickness was determined to be 29 nm. Most importantly, TEM analysis indicated the presence of PHEMA_30_-*b*-PBzMA_291_ worms (Fig. 4b), suggesting that the corresponding 15% w/w free-standing ionogel was formed as a result of the presence of worm-like micelles.^87^ The presence of worms was further evidenced by fitting the background-subtracted SAXS data for PHEMA_30_-*b*-PBzMA_291_ to a worm-like micelle model,^78^ which indicated a mean worm thickness of 43 nm and a worm length of ∼150 nm. In addition to the worm-like micelle model, fitting the SAXS data for this 1% w/w PHEMA_30_-*b*-PBzMA_291_ dispersion required a minor population (17.4% v/v) of spherical nanoparticles with 21 nm in diameter. SAXS data obtained for PHEMA_30_-*b*-PBzMA_146_ nano-objects suggested the presence of aggregated particles as indicated by the significant upturn in scattering intensity at low *q* values. Nevertheless, this background-subtracted scattering pattern was well fitted to a spherical micelle model^78^ that indicated the presence of spheres with a mean diameter of 20 nm, with the upturn at low *q* being represented by an additional power-law relationship. DLS analysis of these nanoparticles also supports the presence of aggregates, with a clear bimodal distribution of smaller particles with similar diameters to those observed by SAXS and larger aggregates between approximately 100 nm and 5 μm in diameter. As a result of this bimodal and broad distribution, the mean DLS diameter was 47 nm with a high PDI value (0.85). TEM analysis also appears to support these observations, however high quality TEM images were difficult to obtain (see Fig. S5†). Finally, SAXS data for a 1% w/w PHEMA_30_-*b*-PBzMA_49_ in [EMIM][DCA] were obtained, which confirmed the presence of molecularly dissolved block copolymer chains. This supports the detailed kinetic study of this PISA formulation (see Fig. 1) that identified the critical PBzMA DP for self-assembly in [EMIM][DCA] as approximately 72. Indeed these data were well fitted to a Gaussian chain model,^77^ indicating polymer chains with a radius of gyration of 2.9 nm. In addition to the four datasets presented in Fig. 4c, SAXS analysis was conducted on all PHEMA_30_-*b*-PBzMA_*y*_ samples and fitted to appropriate models (see Fig. S6, S27 and Table S3†), further confirming that this new PISA formulation yields nanoparticle dispersions containing spheres, worms and vesicles in [EMIM][DCA] ionic liquid.

### Rheological studies of block copolymer worm ionogels

**Fig. 5:**
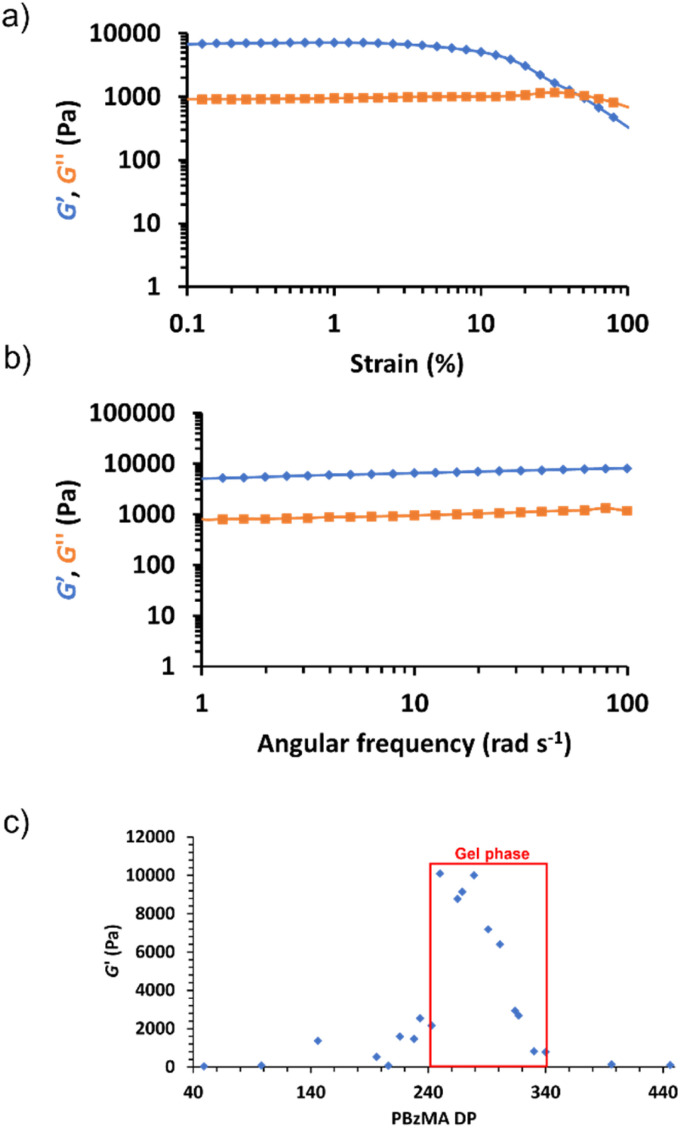
(a) Representative strain sweep data obtained for the 15% w/w PHEMA_30_-*b*-PBzMA_291_ gel at a fixed angular frequency of 6.28 rad s^−1^ and 25 °C. (b) Representative angular frequency sweep data obtained for the 15% w/w PHEMA_30_-*b*-PBzMA_291_ gel at a fixed shear strain of 1.0% and 25 °C. (c) Initial *G*′ *vs.* PBzMA DP for the PHEMA_30_-*b*-PBzMA_*y*_ series at 15% w/w, at a fixed angular frequency of 6.28 rad s^−1^, shear strain of 1.0% and 25 °C. The region outlined in red denotes the gel range based on inversion tests.

Rheological studies of all 15% w/w PHEMA_30_-*b*-PBzMA_*y*_ dispersions were conducted to assess their viscoelastic properties. Specifically, angular frequency and strain sweeps were conducted (see Fig. S28–S48†). The storage modulus, *G*′, obtained at 1% strain and an angular frequency of 6.28 rad s^−1^ for each nanoparticle dispersion was plotted as a function of PBzMA DP as shown in Fig. 5. Samples with PBzMA DP < 228 exhibited a relatively low initial *G*′ (<2500 Pa), which coincides with their physical form of transparent free-flowing liquids. SAXS patterns for dispersions in this PBzMA DP range show that morphologies change from dissolved chains when PBzMA < 100 (see Fig. 4c) to mixtures of spheres and worms for PBzMA DPs between 96 and 228. The *G*′ value increased from ∼2500 Pa to ∼10 000 Pa as the PBzMA DP was increased from 228 to 291, which corresponds to the DP range that forms free-standing gels (see Fig. 2 and 5c). Generally, this is in good agreement with fitting corresponding SAXS data which indicated the presence of worm-like particles, often with a small population of spheres, with samples that exhibited the highest *G*′ value possessing the highest proportion of worms. The presence of more worm-like nanoparticles most likely results in gels with increased *G*′ as a result of an increase in the number of inter-worm contacts and thus the formation of a more extended percolating network.^72^ At PBzMA DPs between 314 and 330, *G*′ broadly decreases from approximately 7000 Pa to 1000 Pa, and fitting SAXS data indicates a substantial decrease in the proportion of worm-like structures alongside a considerable increase in the proportion of vesicles. This coincides with the physical appearance of these dispersions changing from free-standing gels to free-flowing turbid liquids over this PBzMA DP range. At PBzMA DPs > 330, *G*′ decreases from approximately 1000 Pa to a limiting value of ∼100 Pa, and SAXS fittings confirm the presence of vesicular morphologies only. Additionally, oscillatory rheology studies of each free-standing gel sample confirmed their linear viscoelastic behaviour and relatively frequency-independent variation of *G*′ (Fig. 5a and b for representative data and Fig. S28–S48† for all data), indicating the generation of ‘true’ gels.^80^

**Fig. 6:**
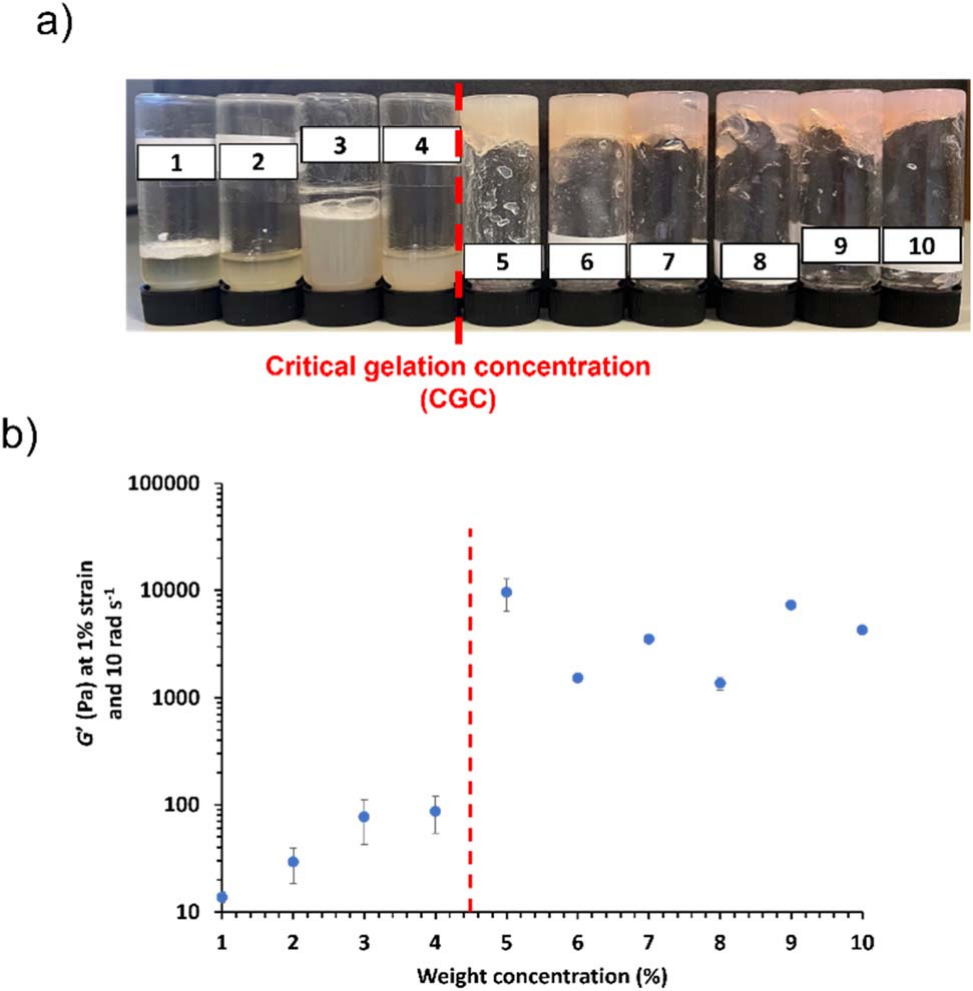
(a) Digital images of critical gelation concentration (CGC) screenings conducted for the synthesis of PHEMA_30_-*b*-PBzMA_291_ between 1% w/w and 10% w/w. (b) *G*′ *vs.* w/w% solids of each gel at a fixed angular frequency of 10 rad s^−1^, 1% strain and 25 °C. Vertical red dashed lines denote the CGC.

In order to maximise electrochemical properties whilst still achieving ionogel formation, it is important to reduce the polymer content. In order to assess the minimum copolymer concentration required to generate a worm ionogel for this formulation, commonly referred to as the critical gel concentration (CGC), a series of targeted PHEMA_30_-*b*-PBzMA_300_ block copolymers were synthesised at copolymer concentrations between 10% w/w and 1% w/w. This target composition was selected for CGC studies since it lies near the centre of the gel phase indicated in Fig. 2 and has the highest proportion of worms as confirmed by SAXS (82.6% v/v worms) (Fig. S19 and Table S3†). Free-standing gels are no longer formed at copolymer concentrations where there are insufficient worm contacts to form a physically crosslinked network, thus yielding free-flowing fluids.^72^ Based on visual observations and tube inversion tests, the CGC for these block copolymer worms was determined to be >4% w/w (see Fig. 6a). This was further evidenced when monitoring the *G*′ of each dispersion (see Fig. 6b), which highlights a significant decrease in *G*′ for copolymer concentrations ≤4% w/w. Importantly, angular frequency sweeps confirmed that *G*′ > *G*′′ for worm dispersions at copolymer concentrations above this observed CGC (see Fig. S49–S58†), as expected.

### Thermal and electrochemical properties of block copolymer worm ionogels

Thermogravimetric analysis (TGA) was used to investigate the thermal stability of selected samples relative to [EMIM][DCA] (Fig. S59†). [EMIM][DCA] appeared to have an onset degradation temperature of approximately 305 °C, which is similar to previously reported studies.^88^ Similarly, the 15% w/w PHEMA_30_-*b*-PBzMA_291_ ionogel also appeared to have a onset degradation temperature of approximately 305 °C, showing that the polymer content in the ionogel has minimal effect on the degradation temperature thus demonstrating that this formulation has good short term thermal stability, an important parameter for future polymer gel electrolytes. In order to further understand this, bulk PHEMA_30_-*b*-PBzMA_293_ was analysed by TGA under the same conditions (Fig. S59†). This bulk block copolymer appeared to have a lower degradation temperature of approximately 230 °C, indicating that the thermal stability of the polymer is potentially improved as a result of the presence of the IL to form the ionogel.

**Fig. 7:**
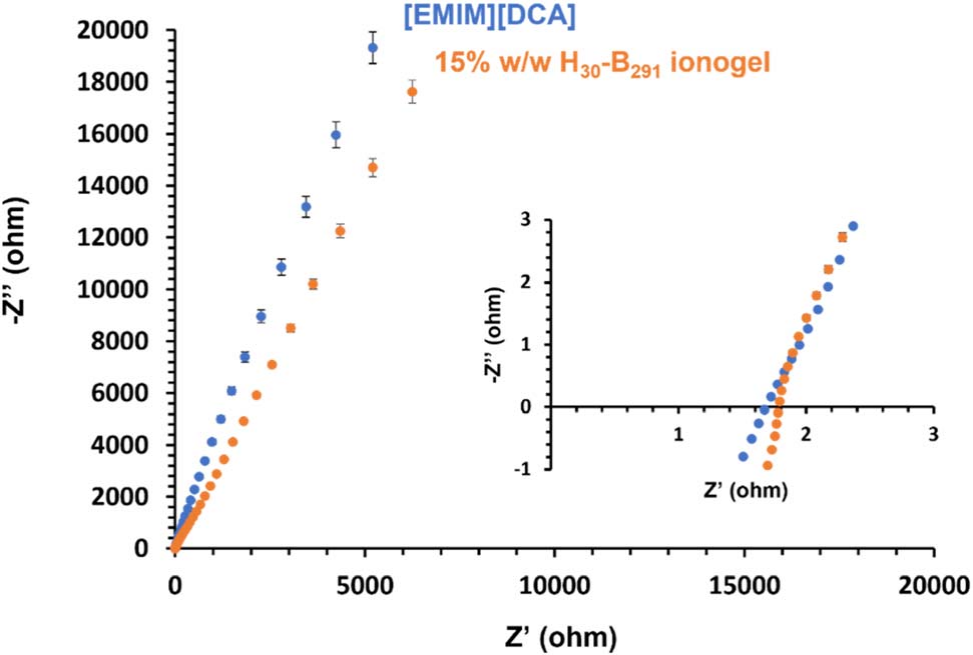
Nyquist plots obtained for [EMIM][DCA] (blue) and PHEMA_30_-*b*-PBzMA_291_ (orange) *via* electrochemical impedance spectroscopy. The inset graph shows the magnified low *Z*′ region to better indicate the *x*-intercept used to determine bulk resistance.

Electrochemical impedance spectroscopy (EIS) was used to compare the bulk resistance of block copolymer worm ionogels and the ionic liquid alone. The bulk resistance was obtained by generating Nyquist plots (Fig. 7). EIS data obtained showed that the ionic liquid, [EMIM][DCA], had a marginally lower bulk resistance (1.67 ± 0.04 Ω) than that of the representative worm ionogel (PHEMA_30_-*b*-PBzMA_291_) which had a bulk resistance of 1.79 ± 0.15 Ω. When considering the standard deviation in these values, which were determined from repeating results in triplicate, the differences in bulk resistance of the ionogel and [EMIM][DCA] were deemed to be statistically insignificant, meaning that the presence of 15% w/w non-ionic PHEMA_30_-*b*-PBzMA_291_ did not negatively impact the electrochemical properties of the IL. This is important and suggests that these gel electrolytes formed using this new approach have great potential for potential applications in the energy storage sector.

## Conclusions

In summary, a series of PHEMA_30_-*b*-PBzMA_*y*_ block copolymers were synthesised *via* RAFT dispersion polymerisation of benzyl methacrylate in [EMIM][DCA] at 70 °C and 15% w/w solids. The resulting dispersions yielded a range of block copolymer nano-objects including spheres, worms and vesicles as confirmed by a combination of DLS, SAXS and TEM analyses. High monomer conversions were achieved within 120 minutes of these polymerisation-induced self-assembly (PISA) syntheses and GPC analysis demonstrated good control of the polymerisation when targeting a range of PBzMA DPs. Most notably, this formulation enables, for the first time, the *in situ* generation of free-standing worm ionogels during PISA. Moreover, we demonstrate that the minimum copolymer concentration at which worm ionogels are observed for this formulation, the critical gelation concentration (CGC), is >4% w/w. Such a low required copolymer concentration, coupled with the fact that these worm ionogels demonstrate comparable electrochemical properties and thermal stability to those of [EMIM][DCA] alone, showcases this approach's promise in future energy storage applications. In principle, this approach facilitates a more convenient and facile route to generating ionic liquid-based gel electrolytes owing to the lack of requirement for additional co-solvent and post-polymerisation or purification processes.

## Data availability

Data for this paper, including polymer and nanoparticle characterisation data, small-angle X-ray scattering fitting and models, oscillatory rheology data and electrochemical impedance spectroscopy data, are available in the ESI.†

## Author contributions

The authors contributed to this work in the following ways: Conceptualisation (M. J. D.); Formal analysis (T. J. N.); Funding acquisition (M. J. D.); Investigation (G. L. M., M. L., J. H., Y. H., S. D. W.); Methodology (G. L. M., M. J. D.); Project administration (M. J. D.); Supervision (P. D. T., M. J. D.); Validation (G. L. M.); Visualisation (G. L. M., M. J. D.); Writing – original draft (G. L. M., M. J. D.); Writing – review & editing (G. L. M., M. L., T. J. N., J. H., Y. H., S. D. W., P. D. T., M. J. D.).

## Conflicts of interest

There are no conflicts to declare.

## Supplementary Material

SC-015-D3SC06717C-s001
