# Supplementary material for: Block copolymer synthesis in ionic liquid via polymerisation-induced self-assembly: a convenient route to gel electrolytes
Source: Chem Sci. 2024 Feb 13;15(12):4416–26. doi: 10.1039/d3sc06717c (PMC10952082; doi:10.1039/d3sc06717c)
Supplement: SC-015-D3SC06717C-s001 [file SC-015-D3SC06717C-s001.pdf]

Electronic Supplementary Material (ESI) for Chemical Science.
This journal is © The Royal Society of Chemistry 2024

Supporting Information for:

Block copolymer synthesis in ionic liquid via polymerisation-induced self-assembly: A convenient route to gel electrolytes

*Georgia L. Maitland,[a] Mingyu Liu,[a] Thomas J. Neal,[b] James Hammerton,[a] Yisong Han,[c] Stephen D. Worrall,[a] Paul D. Topham,[a] and Matthew J. Derry[a,]**

Contents

Determination of HEMA monomer conversion in PHEMA macro-CTA synthesis 2
Determination of PHEMA macro-CTA degree of polymerisation 3
Determination of BzMA monomer conversion in PISA syntheses 4
Summary of block copolymer characterisation 5
Additional dynamic light scattering data 7
Additional transmission electron microscopy images 7
Small-angle X-ray scattering 8
Additional oscillatory rheology data 26
Thermogravimetric analysis 41

Determination of HEMA monomer conversion in PHEMA macro-CTA synthesis

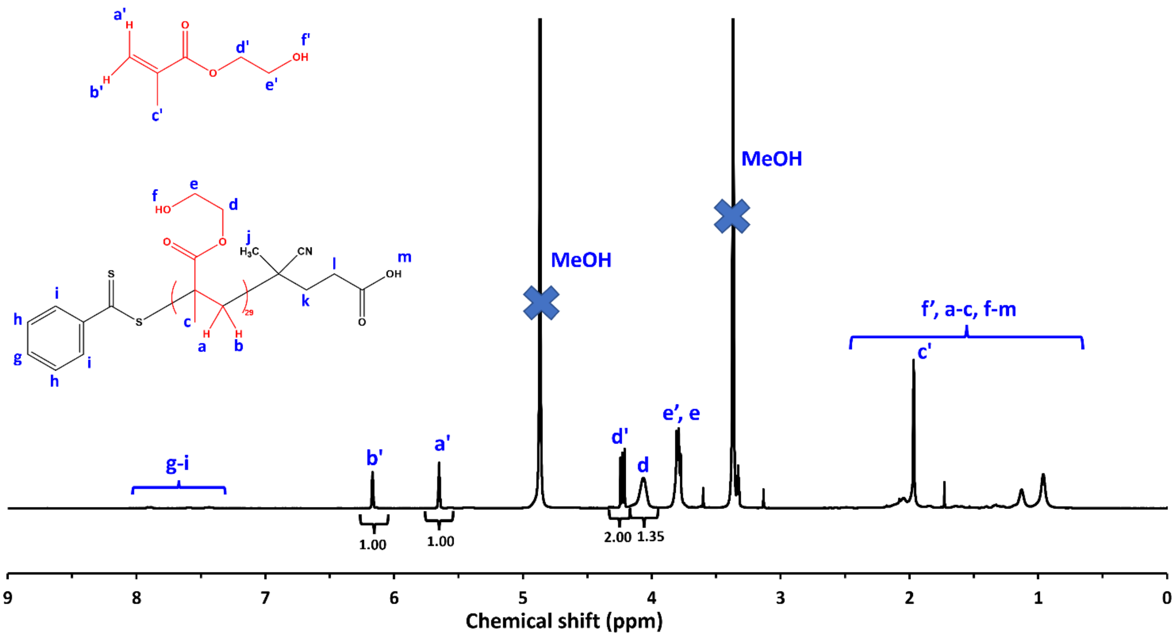

Figure S1. ^{1}H NMR spectrum of crude PHEMA macro-CTA in MeOH-d_4.

I_m = [Integral (d')] = 2H

I_p = [Integral (d)]

$$\% \text{ Conversion} = \frac{I_p}{I_m + I_p} \times 100\% = \frac{1.35}{2.00 + 1.35} \times 100\% = \mathbf{40\%}$$

Determination of PHEMA macro-CTA degree of polymerisation

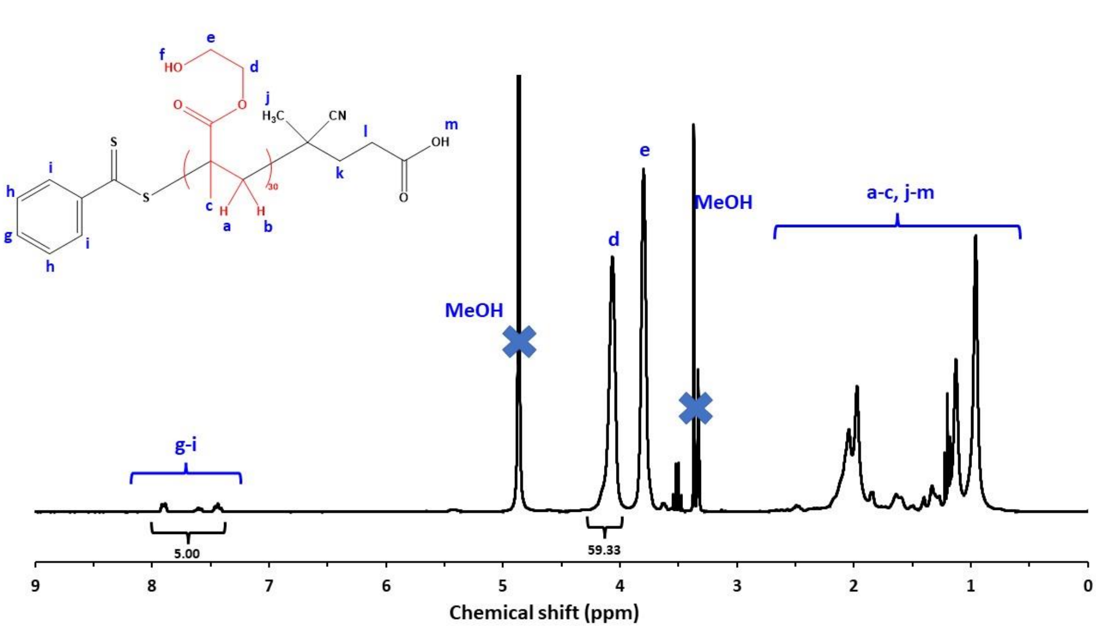

Figure S2. ^{1}H NMR spectrum of purified $PHEMA_{30}$ macro-CTA in MeOH-d_4.

[Integral (g-i)] = 5H

$$PHEMA\ DP = \frac{[Integral\ (d)]}{2} = \frac{59.33}{2} = \mathbf{30}$$

Determination of BzMA monomer conversion in PISA syntheses

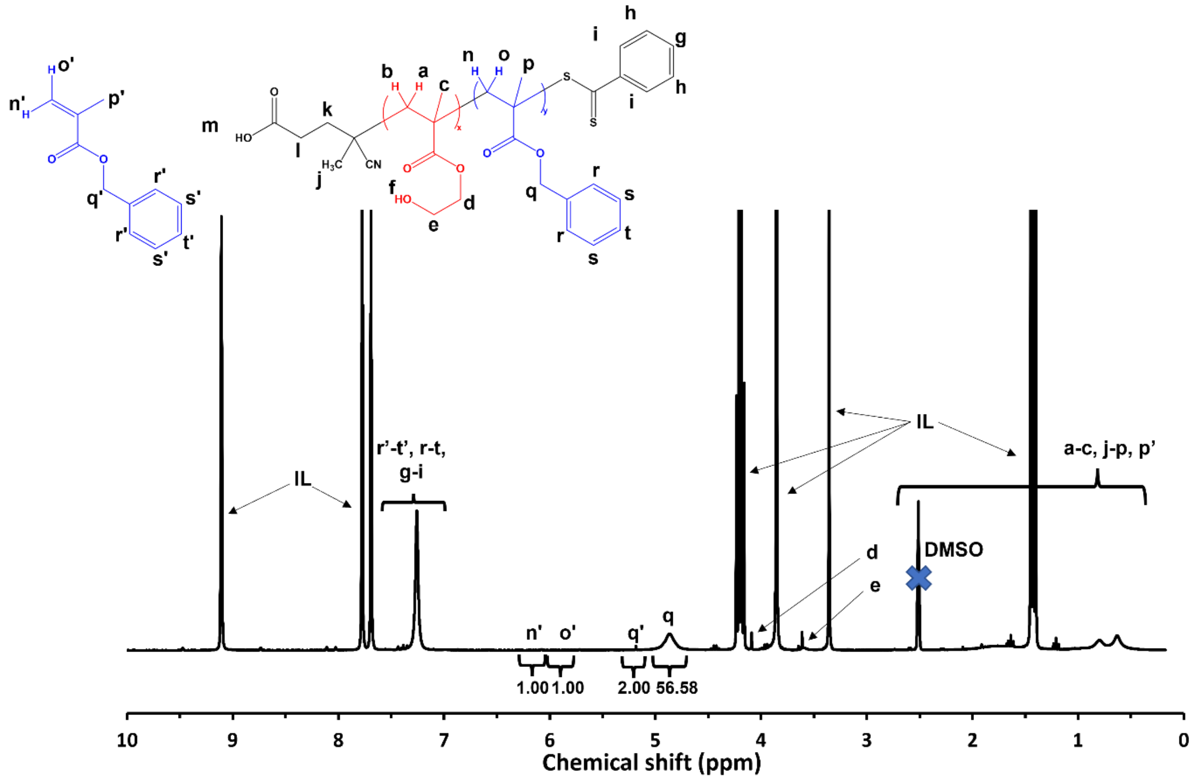

Figure S3. Representative ^{1}H NMR spectrum of crude PHEMA$_{30}$-*b*-PBzMA$_y$ in DMSO-d$_6$.

Example calculation:

I_m = [Integral (d')] = 2H

I_p = [Integral (d)]

$$\% \text{ Conversion} = \frac{I_p}{I_m + I_p} \times 100\% = \frac{56.58}{2.00 + 56.58} \times 100\% = \mathbf{97\%}$$

Summary of block copolymer characterisation

Table S1. Summary of targeted copolymer composition, BzMA conversion, actual copolymer composition, theoretical M_n, GPC M_n and M_w/M_n, and DLS diameter and PDI for the series of PHEMA$_{30}$-*b*-PBzMA$_y$ diblock copolymers prepared by RAFT dispersion polymerisation of BzMA in [EMIM][DCA] at 70 °C and 15% w/w, using AIBN initiator ([PHEMA$_{30}$ macro-CTA]/[AIBN] molar ratio = 5.0). PHPMA$_{30}$-*b*-PBzMA$_y$ is denoted as H$_{30}$-B$_y$ for brevity.

		^{1}H NMR spectroscopy		DMF GPC		DLS	
**Target composition**	**BzMA conversion (%)**	**Actual composition**	$M_{n,th}$ **(g mol^{-1})**	M_n **(g mol^{-1})**	M_w/M_n	**Diameter (nm)**	**PDI**
H$_{30}$	-	H$_{30}$	4,184	8,000	1.24	-	-
H$_{30}$-B$_{50}$	97	H$_{30}$-B$_{49}$	12,688	18,100	1.24	-	-
H$_{30}$-B$_{100}$	98	H$_{30}$-B$_{98}$	21,322	24,900	1.21	39	0.98
H$_{30}$-B$_{150}$	97	H$_{30}$-B$_{146}$	29,780	24,000	1.20	47	0.85
H$_{30}$-B$_{200}$	98	H$_{30}$-B$_{196}$	38,591	42,500	1.24	90	0.32
H$_{30}$-B$_{210}$	98	H$_{30}$-B$_{201}$	40,353	40,600	1.21	137	1.00
H$_{30}$-B$_{220}$	98	H$_{30}$-B$_{216}$	42,115	40,000	1.34	100	0.67
H$_{30}$-B$_{230}$	99	H$_{30}$-B$_{228}$	44,230	41,400	1.39	134	0.57
H$_{30}$-B$_{240}$	97	H$_{30}$-B$_{233}$	45,111	45,500	1.30	88	0.84
H$_{30}$-B$_{250}$	97	H$_{30}$-B$_{240}$	46,873	47,800	1.23	153	0.14
H$_{30}$-B$_{260}$	96	H$_{30}$-B$_{250}$	48,107	46,700	1.30	129	0.04
H$_{30}$-B$_{270}$	98	H$_{30}$-B$_{262}$	50,750	50,700	1.34	210	0.24
H$_{30}$-B$_{280}$	96	H$_{30}$-B$_{269}$	51,455	53,100	1.26	221	0.11
H$_{30}$-B$_{290}$	96	H$_{30}$-B$_{279}$	53,217	48,700	1.43	134	0.13
H$_{30}$-B$_{300}$	97	H$_{30}$-B$_{291}$	55,331	52,900	1.31	238	0.06
H$_{30}$-B$_{310}$	98	H$_{30}$-B$_{301}$	57,093	56,800	1.33	31	0.60
H$_{30}$-B$_{320}$	98	H$_{30}$-B$_{314}$	59,384	57,000	1.29	146	0.28
H$_{30}$-B$_{330}$	96	H$_{30}$-B$_{317}$	59,913	60,200	1.34	145	0.47
H$_{30}$-B$_{340}$	97	H$_{30}$-B$_{330}$	62,203	57,500	1.36	245	0.07
H$_{30}$-B$_{350}$	97	H$_{30}$-B$_{340}$	63,966	57,300	1.49	356	0.07
H$_{30}$-B$_{400}$	99	H$_{30}$-B$_{396}$	73,833	66,700	1.43	272	0.08
H$_{30}$-B$_{450}$	99	H$_{30}$-B$_{446}$	82,644	67,200	1.53	273	0.08
H$_{30}$-B$_{500}$	98	H$_{30}$-B$_{490}$	90,397	79,900	1.56	692	0.58

Table S2. Summary of targeted copolymer composition, BzMA conversion, actual copolymer composition, theoretical M_n, GPC M_n and M_w/M_n, and DLS diameter and PDI for targeted PHEMA$_{30}$-*b*-PBzMA$_{300}$ diblock copolymers prepared by RAFT dispersion polymerisation of BzMA in [EMIM][DCA] at 70 °C and various copolymer concentrations, using AIBN initiator ([PHEMA$_{30}$ macro-CTA]/[AIBN] molar ratio = 5.0). PHPMA$_{30}$-*b*-PBzMA$_y$ is denoted as H$_{30}$-B$_y$ for brevity.

		**^{1}H NMR spectroscopy**		**DMF GPC**		**DLS**	
**Copolymer concentration (% w/w)**	**BzMA conversion (%)**	**Actual composition**	**$M_{n,th}$ (g mol^{-1})**	**M_n (g mol^{-1})**	**M_w/M_n**	**Diameter (nm)**	**PDI**
15	97	H$_{30}$-B$_{291}$	55,331	52,900	1.31	238	0.06
10	90	H$_{30}$-B$_{270}$	51,761	53,200	1.36	500	0.36
9	94	H$_{30}$-B$_{282}$	53,875	56,100	1.43	324	0.27
8	89	H$_{30}$-B$_{267}$	51,232	53,100	1.32	361	0.30
7	89	H$_{30}$-B$_{267}$	51,232	50,200	1.47	317	0.41
6	98	H$_{30}$-B$_{294}$	55,990	53,200	2.12	200	0.28
5	97	H$_{30}$-B$_{291}$	55,461	69,500	1.67	770	0.76
4	98	H$_{30}$-B$_{294}$	55,990	53,600	1.94	-	-
3	92	H$_{30}$-B$_{276}$	52,818	63,000	1.39	-	-
2	99	H$_{30}$-B$_{297}$	56,518	43,600	1.67	-	-
1	95	H$_{30}$-B$_{285}$	54,404	33,500	1.49	-	-

Additional dynamic light scattering data

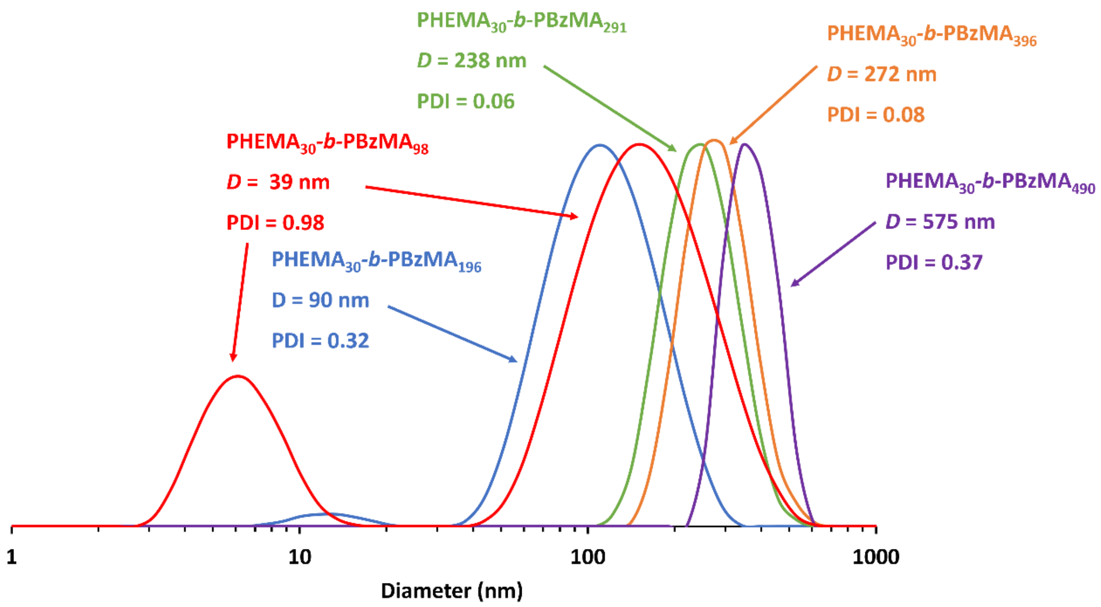

Figure S4. DLS traces obtained of selected 0.15% w/w dispersions block copolymer series.

Additional transmission electron microscopy images

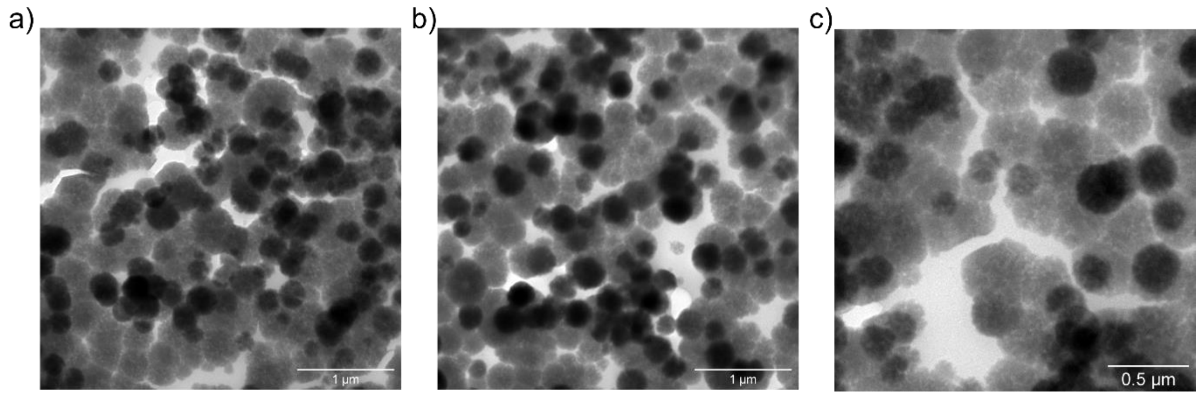

Figure S5. TEM images obtained for a 15% w/w dispersion of PHEMA$_{30}$-*b*-PBzMA$_{146}$.

Small-angle X-ray scattering

Programming tools within the Irena SAS Igor Pro macros[1] were used to implement the scattering models.

In general, the intensity of X-rays scattered by a dispersion of nano-objects [as represented by the scattering cross-section per unit sample volume, $\frac{d\Sigma}{d\Omega}(q)$] can be expressed as:

$$\frac{d\Sigma}{d\Omega}(q) = NS(q)\int_0^\infty \dots \int_0^\infty F(q, r_1, \dots, r_k)^2 \Psi(r_1, \dots, r_k) dr_1, \dots, dr_k \quad \text{(S1)}$$

where $F(q, r_1, \dots, r_k)$ is the form factor, $r_1, \dots, r_k$ is a set of k parameters describing the structural morphology, $\Psi(r_1, \dots, r_k)$ is the distribution function, $S(q)$ is the structure factor and N is the number density of nano-objects per unit volume expressed as:

$$N = \frac{\varphi}{\int_0^\infty \dots \int_0^\infty V(r_1, \dots, r_k)\Psi(r_1, \dots, r_k) dr_1, \dots, dr_k} \quad \text{(S2)}$$

where $V(r_1, \dots, r_k)$ is the volume of the nano-object and φ is its volume fraction within the dispersion. It is assumed that $S(q) = 1$ at the sufficiently low copolymer concentrations used in this study (1.0% w/w).

Spherical micelle model

The spherical micelle form factor for Equation S1 is given by[2]

$$F_{s_{mic}}(q) = N_s{}^2\beta_s{}^2A_s{}^2(q, R_s) + N_s\beta_c{}^2F_c(q, R_g) + N_s(N_s - 1)\beta_c{}^2A_c{}^2(q) + 2N_s{}^2\beta_s\beta_cA_s(q, R_s)A_c(q) \quad \text{(S3)}$$

where R_s is the volume-average sphere core radius and R_g is the radius of gyration of the coronal steric stabilizer block (in this case, $PHEMA_{30}$). The X-ray scattering length contrasts for the core and corona blocks are given by $\beta_s = V_s(\xi_s - \xi_{sol})$ and $\beta_c = V_c(\xi_c - \xi_{sol})$ respectively. Here, ξ_s, ξ_c and ξ_{sol} are the X-ray scattering length densities of the core block (ξ_{PBzMA}= 10.41 x 10^{10} cm^{-2}), corona block (ξ_{PHEMA}= 11.50 x 10^{10} cm^{-2}) and [EMIM][DCA] solvent (ξ_{sol}= 9.90 x 10^{10} cm^{-2}), respectively. V_s and V_c are the volumes of the core block (V_{PBzMA}) and the corona block (V_{PHEMA}), respectively. The sphere form factor amplitude is used for the amplitude of the core self-term:

$$A_c(q, R_s) = \Phi(qR_s)\exp\left(-\frac{q^2\sigma^2}{2}\right) \quad \text{(S4)}$$

where $\Phi(qR_s) = \frac{3[\sin(qR_s) - qR_s\cos(qR_s)]}{(qR_s)^3}$. A sigmoidal interface between the two blocks was assumed for the spherical micelle form factor (Equation S3). This is described by the exponent term with a width σ accounting for a decaying scattering length density at the micellar interface. This σ value was fixed at 2.5 during fitting.

The form factor amplitude of the spherical micelle corona is:

$$A_c(q) = \frac{\int_{R_s}^{R_s+2s} \mu_c(r)\frac{\sin(qr)}{qr}r^2dr}{\int_{R_s}^{R_s+2s} \mu_c(r)r^2dr}exp\left(-\frac{q^2\sigma^2}{2}\right) \quad \text{(S5)}$$

The radial profile, $\mu_c(r)$, can be expressed by a linear combination of two cubic b splines, with two fitting parameters s and a corresponding to the width of the profile and the weight coefficient respectively. This information can be found elsewhere,[3, 4]as can the approximate integrated form of Equation S5. The self-correlation term for the coronal block is given by the Debye function:

$$F_c(q, R_g) = \frac{2\left[\exp\left(-q^2{R_g}^2\right) - 1 + q^2{R_g}^2\right]}{q^4{R_g}^4} \quad \text{(S6)}$$

where R_g is the radius of gyration of the PHEMA coronal block. In all cases R_g was fixed to be 1.4 nm, which is estimated by assuming the total contour length of $PHMEA_{30}$ is 7.66 nm (30 × 0.255 nm, where 0.225 nm is the contour length of one HEMA monomer unit with two C-C bonds in all-trans conformation). Given a mean Kuhn length of 1.53 nm, based on the known literature value for poly(methyl methacrylate)[5], an estimated unperturbed R_g of 1.4 nm is determined using R_g = $(7.66 \times 1.53/6)^{0.5}$.

The aggregation number, N_s, of the spherical micelle is given by:

$$N_s = (1 - x_{sol})\frac{\frac{4}{3}\pi{R_s}^3}{V_s} \quad \text{(S7)}$$

where x_{sol} is the volume fraction of solvent within the PBzMA micelle cores, which was found to be zero in all cases. A polydispersity for one parameter (R_s) is assumed for the micelle model, which is described by a Gaussian distribution. Thus, the polydispersity function in Equation S1 can be represented as:

$$\Psi(r_1) = \frac{1}{\sqrt{2\pi\sigma_{Rs}{}^2}} exp\left(-\frac{(r_1 - R_s)^2}{2\sigma_{Rs}{}^2}\right) \quad \text{(S8)}$$

where σ_{Rs} is the standard deviation for R_s. In accordance with Equation S2, the number density per unit volume for the micelle model is expressed as:

$$N = \frac{\varphi}{\int_0^\infty V(r_1)\Psi(r_1)dr_1} \quad \text{(S9)}$$

where φ is the total volume fraction of copolymer in the spherical micelles and $V(r_1)$ is the total volume of copolymer within a spherical micelle $[V(r_1) = (V_s + V_c)N_s(r_1)]$.

Worm-like micelle model

The worm-like micelle form factor for Equation S1 is given by:

$$F_{w\_mic}(q) = N_w{}^2\beta_s{}^2F_{sw}(q) + N_w\beta_c{}^2F_c(q, R_g) + N_w(N_w - 1)\beta_c{}^2S_{cc}(q) + 2N_w{}^2\beta_s\beta_cS_{sc}(q) \quad \text{(S10)}$$

where all the parameters are the same as those described in the spherical micelle model (Equation S3), unless stated otherwise.

The self-correlation term for the worm core cross-sectional volume-average radius R_w is:

$$F_{sw}(q) = F_{worm}(q, L_w, b_w)A_{CS_{worm}}{}^2(q, R_w) \quad \text{(S11)}$$

where

$$A_{CS_{worm}}{}^2(q, R_w) = \left[2\frac{J_1(qR_w)}{qR_w}\right]^2 \quad \text{(S12)}$$

and J_1 is the first-order Bessel function of the first kind, and a form factor $F_{worm}(q, L_w, b_w)$ for self-avoiding semi-flexible chains represents the worm-like micelles, where b_w is the Kuhn length and L_w is the mean contour length. A complete expression for the chain form factor can be found elsewhere.[6]

The mean aggregation number of the worm-like micelle, N_w, is given by:

$$N_w = (1 - x_{sol})\frac{\pi {R_w}^2 L_w}{V_s} \tag{S13}$$

where x_{sol} is the volume fraction of solvent within the worm-like micelle cores, which was found to be zero in all cases. The possible presence of semi-spherical caps at both ends of each worm is neglected in this form factor.

A polydispersity for one parameter (R_w) is assumed for the micelle model, which is described by a Gaussian distribution. Thus, the polydispersity function in Equation S1 can be represented as:

$$\Psi(r_1) = \frac{1}{\sqrt{2\pi {\sigma_{R_\mathrm{w}}}^2}} exp\left(-\frac{(r_1 - R_\mathrm{w})^2}{2{\sigma_{R_\mathrm{w}}}^2}\right) \tag{S14}$$

where σ_{R_w} is the standard deviation for R_w. In accordance with Equation S2, the number density per unit volume for the worm-like micelle model is expressed as:

$$N = \frac{\varphi}{\int_0^\infty V(r_1)\Psi(r_1)dr_1} \tag{S15}$$

where φ is the total volume fraction of copolymer in the worm-like micelles and $V(r_1)$ is the total volume of copolymer in a worm-like micelle $[V(r_1) = (V_\mathrm{s} + V_\mathrm{c})N_\mathrm{w}(r_1)]$.

Vesicle model

The vesicle form factor in Equation S1 is expressed as:[7]

$$F_{ves}(q) = {N_v}^2{\beta_m}^2{A_m}^2(q) + N_v{\beta_{vc}}^2F_c(q,R_g) + N_v(N_v - 1){\beta_{vc}}^2{A_{vc}}^2(q) + 2{N_v}^2\beta_m\beta_{vc}A_m(q)A_{vc}(q) \tag{S16}$$

where all the parameters are the same as in the spherical micelle model (see Equation S3) unless stated otherwise.

The amplitude of the membrane self-term is:

$$A_m(q) = \frac{V_{out}\varphi(qR_{out}) - V_{in}\varphi(qR_{in})}{V_{out} - V_{in}} exp\left(-\frac{q^2{\sigma_{in}}^2}{2}\right) \tag{S17}$$

where $R_{in} = R_m - \frac{1}{2}T_m$ is the inner radius of the membrane, $R_{out} = R_m + \frac{1}{2}T_m$ is the outer radius of the membrane (R_{m} is the radius from the centre of the vesicle to the centre of the membrane), $V_{in} = \frac{4}{3}\pi {R_{in}}^3$ and $V_{out} = \frac{4}{3}\pi {R_{out}}^3$. It should be noted that Equation S16 differs subtly from the original work in which it was first described.[7]The exponent term in Equation S17 represents a sigmoidal interface between the blocks, with a width σ_{in} accounting for a decaying scattering length density at the membrane surface. The value of σ_{in} was fixed at 2.5 during fitting. The mean vesicle aggregation number, N_v, is given by:

$$N_v = (1 - x_{sol})\frac{V_{out} - V_{in}}{V_m} \quad \text{(S18)}$$

where x_{sol} is the volume fraction of solvent within the vesicle membrane, which was found to be zero in all cases. Assuming that there is no penetration of the solvophilic coronal blocks into the solvophobic membrane, the amplitude of the vesicle corona self-term is expressed as:

$$A_{vc}(q) = \Psi(qR_g)\frac{1}{2}\left[\frac{sin[q(R_{out} + R_g)]}{q(R_{out} + R_g)} + \frac{\sin\,[q(R_{in} - R_g)]}{q(R_{in} - R_g)}\right] \quad \text{(S19)}$$

where the term outside the square brackets is the factor amplitude of the corona block polymer chain such that:

$$\Psi(qR_g) = \frac{1 - \exp\,(-qR_g)}{(qR_g)^2} \quad \text{(S20)}$$

For the vesicle model, it was assumed that two parameters are polydisperse: the radius from the centre of the vesicles to the centre of the membrane and the membrane thickness (denoted R_m and T_m, respectively). Each parameter is considered to have a Gaussian distribution of values, so the polydispersity function in Equation S1 can be expressed in each case as:

$$\Psi(r_1 r_2) = \frac{1}{\sqrt{2\pi {\sigma_{Rs}}^2}} exp\left(-\frac{(r_1 - R_m)^2}{2{\sigma_{Rm}}^2}\right)\frac{1}{\sqrt{2\pi {\sigma_{Tm}}^2}} exp\left(-\frac{(r_1 - T_m)^2}{2{\sigma_{Tm}}^2}\right) \quad \text{(S21)}$$

where σ_{Rm} and σ_{Tm} are the standard deviations for R_m and T_m, respectively. Following Equation S2, the number density per unit volume for the vesicle model is expressed as:

$$N = \frac{\varphi}{\int_0^\infty \int_0^\infty V(r_1, r_2)\,\Psi(r_1, r_2) dr_1 dr_2} \quad \text{(S22)}$$

where φ is the total volume fraction of copolymer in the vesicles and $V(r_1, r_2)$ is the total volume of copolymers in a vesicle $[V(r_1, r_2) = (V_m + V_{vc})N_v(r_1, r_2)]$.

Gaussian chain model

Data for the 1% w/w solution of PHEMA$_{30}$-*b*-PBzMA$_{49}$ were fitted to a Gaussian chain model.[8] Generally, the scattering cross-section per unit sample volume for an individual Gaussian polymer chain can be expressed as:

$$\frac{d\Sigma}{d\Omega}(q) = \varphi(\Delta\xi)^2 V_{\mathrm{mol}} F_{\mathrm{mol}}(q) \qquad \text{(S23)}$$

where V_{mol} is the total molecular volume and $\Delta\xi$ is the excess scattering length density of the copolymer [$\Delta\xi = \xi_{\mathrm{PHEMA-PBzMA}} - \xi_{\mathrm{[EMIM][DCA]}}$ = 0.92 x 10^{-10} cm^{-2}], where the scattering length density of the copolymer, $\xi_{\mathrm{PHEMA-PBzMA}} = \frac{V_{\mathrm{PHEMA}}\xi_{\mathrm{PHEMA}} + V_{\mathrm{PBzMA}}\xi_{\mathrm{PBzMA}}}{V_{\mathrm{PHEMA-PBzMA}}}$ which for PHEMA$_{30}$-*b*-PBzMA$_{49}$ gives $\xi_{\mathrm{PHEMA-PBzMA}}$ = 10.72 x 10^{-10} cm^{-2}, and the scattering length density of [EMIM][DCA], $\xi_{[EMIM][DCA]}$ = 9.90 x 10^{-10} cm^{-2}. The generalized form factor for a Gaussian polymer chain is given by:

$$F_{\mathrm{mol}}(q) = \left[\frac{1}{\upsilon U^{1/(2\upsilon)}}\gamma\left(\frac{1}{2\upsilon}, U\right) - \frac{1}{\upsilon U^{1/\upsilon}}\gamma\left(\frac{1}{\upsilon}, U\right)\right] \qquad \text{(S24)}$$

where the lower incomplete gamma function is $\gamma(s, x) = \int_0^x t^{s-1}\exp(-t)\,dt$ and U is the modified variable:

$$U = (2\upsilon + 1)(2\upsilon + 2)\frac{q^2 {R_{\mathrm{g}_{\mathrm{cop}}}}^2}{6} \qquad \text{(S25)}$$

Here, υ is the extended volume parameter and $R_{\mathrm{g}_{\mathrm{cop}}}$ is the radius of gyration of the copolymer chain.

Table S3. Summary of parameters obtained when fitting SAXS data to appropriate models. $\varphi_{\mathrm{sphere}}$, φ_{worm} and $\varphi_{\mathrm{vesicle}}$ are the volume fraction of spheres, worms and vesicles, respectively. D_{sphere} is the spherical nanoparticle diameter ($D_{\mathrm{sphere}} = 2R_{\mathrm{s}} + 4R_{\mathrm{g}}$, where R_{g} is the radius of gyration of the stabiliser block and R_{s} is the core radius). T_{worm} is the worm thickness ($T_{\mathrm{worm}} = 2R_{\mathrm{w}} + 4R_{\mathrm{g}}$, where R_{w} is the worm core cross-sectional radius). L_{worm} is the worm length. D_{vesicle} is the overall vesicle diameter ($D_{\mathrm{vesicle}} = R_{\mathrm{m}} + T_{\mathrm{m}} + 4R_{\mathrm{g}}$, where R_{m} is the centre of the vesicle to the centre of the membrane and T_{m} is the membrane thickness). $R_{\mathrm{g_{cop}}}$ is the radius of gyration of dissolved copolymer chains. υ is the extended volume parameter. $PHEMA_{30}$-*b*-$PBzMA_{y}$ is denoted as H_{30}-B_{y} for brevity.

	**Spherical micelle model**		**Worm-like micelle model**			**Vesicle model**			**Gaussian chain model**	
**Sample**	$\boldsymbol{\varphi_{\mathrm{sphere}}}$	$\boldsymbol{D_{\mathrm{sphere}}}$ **(nm)**	$\boldsymbol{\varphi_{\mathrm{worm}}}$	$\boldsymbol{T_{\mathrm{worm}}}$ **(nm)**	$\boldsymbol{L_{\mathrm{worm}}}$ **(nm)**	$\boldsymbol{\varphi_{\mathrm{vesicle}}}$	$\boldsymbol{D_{\mathrm{vesicle}}}$ **(nm)**	$\boldsymbol{T_{\mathrm{m}}}$ **(nm)**	$\boldsymbol{R_{\mathrm{g_{cop}}}}$ **(nm)**	$\boldsymbol{\upsilon}$
H_{30}-B_{49}									2.90	0.5
H_{30}-B_{98}	0.0141	20.0								
H_{30}-B_{146}	0.0227	20.4								
H_{30}-B_{196}	0.0066	20.7	0.0124	35.6	140					
H_{30}-B_{201}	0.0118	22.7	0.0085	34.5	256					
H_{30}-B_{216}	0.0160	21.1	0.0068	32.2	600					
H_{30}-B_{228}	0.0084	21.3	0.0123	35.4	212					
H_{30}-B_{233}	0.0127	21.9	0.0080	35.1	156					
H_{30}-B_{240}	0.0036	22.3	0.0121	38.7	159					
H_{30}-B_{250}			0.0050	17.4	600					
H_{30}-B_{265}	0.0052	21.3	0.0223	39.0	212					
H_{30}-B_{269}	0.0020	21.5	0.0127	42.8	216					
H_{30}-B_{279}	0.0054	21.4	0.0111	40.3	197					
H_{30}-B_{291}	0.0017	20.7	0.0079	42.7	149					
H_{30}-B_{301}	0.0172	21.6	0.0056	39.2	240					
H_{30}-B_{314}			0.0012	19.6	100	0.0175	409	24.6		
H_{30}-B_{317}			0.0007	17.8	100	0.0174	440	25.1		
H_{30}-B_{330}			0.0040	59.2	100	0.0155	348	25.4		
H_{30}-B_{340}			0.0008	38.1	104	0.0166	311	26.3		
H_{30}-B_{396}						0.0147	315	29.0		
H_{30}-B_{446}						0.0162	302	31.3		
H_{30}-B_{490}						0.0530	307	33.3		

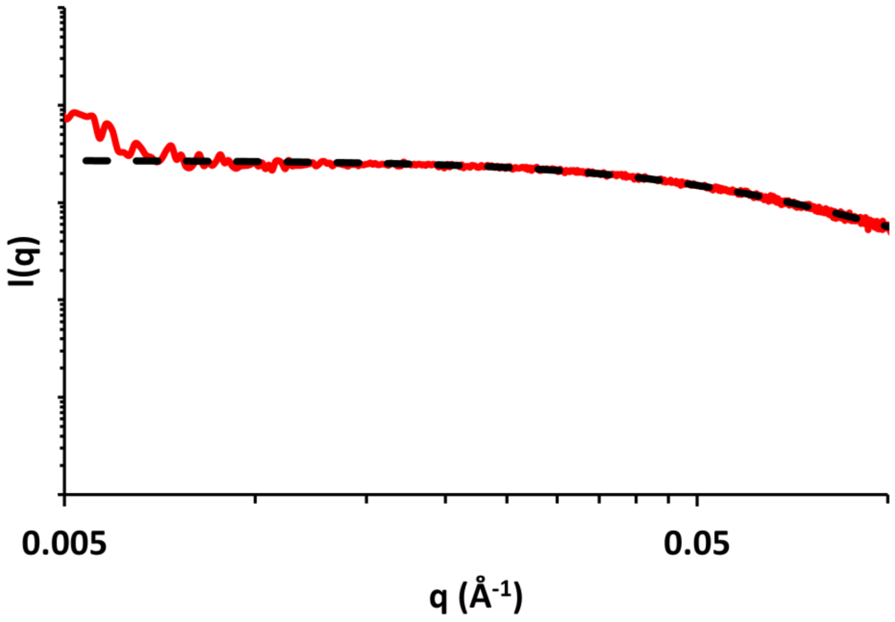

Figure S6. Background-subtracted SAXS data obtained for 1.0% w/w $PHEMA_{30}$-$PBzMA_{49}$ in [EMIM][DCA] at 25 °C. Dashed lines represent the model fit obtained using the Gaussian chain model.

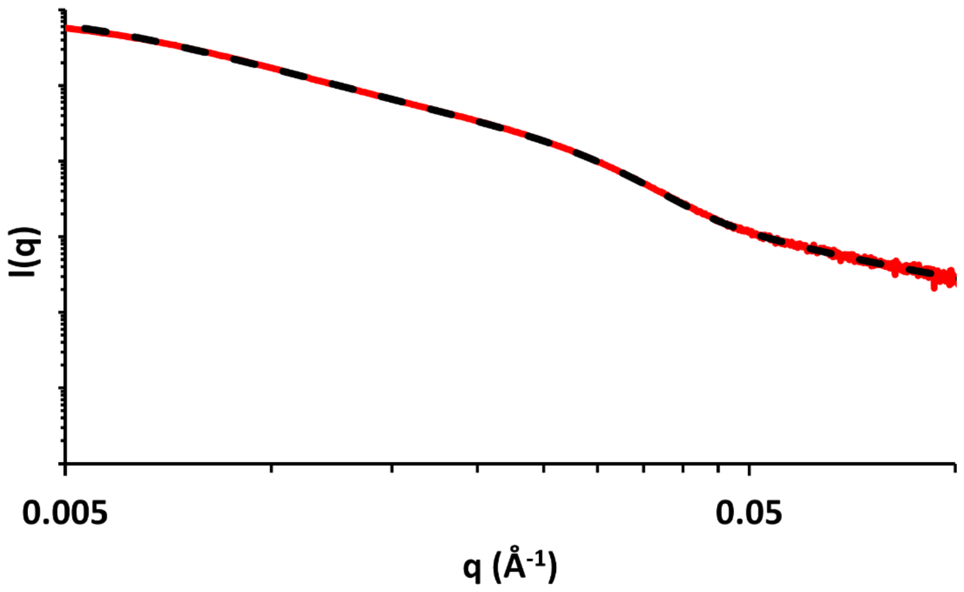

Figure S7. Background-subtracted SAXS data obtained for 1.0% w/w $PHEMA_{30}$-$PBzMA_{98}$ in [EMIM][DCA] at 25 °C. Dashed lines represent the model fit obtained using the spherical micelle model with an additional power law to account for the upturn in scattering at low *q*.

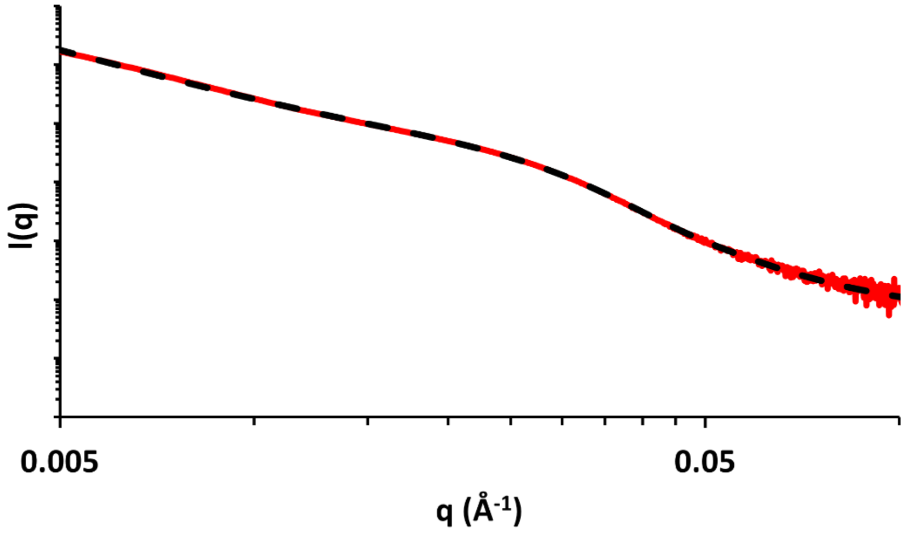

Figure S8. Background-subtracted SAXS data obtained for 1.0% w/w $PHEMA_{30}$-$PBzMA_{146}$ in [EMIM][DCA] at 25 °C. Dashed lines represent the model fit obtained using the spherical micelle model.

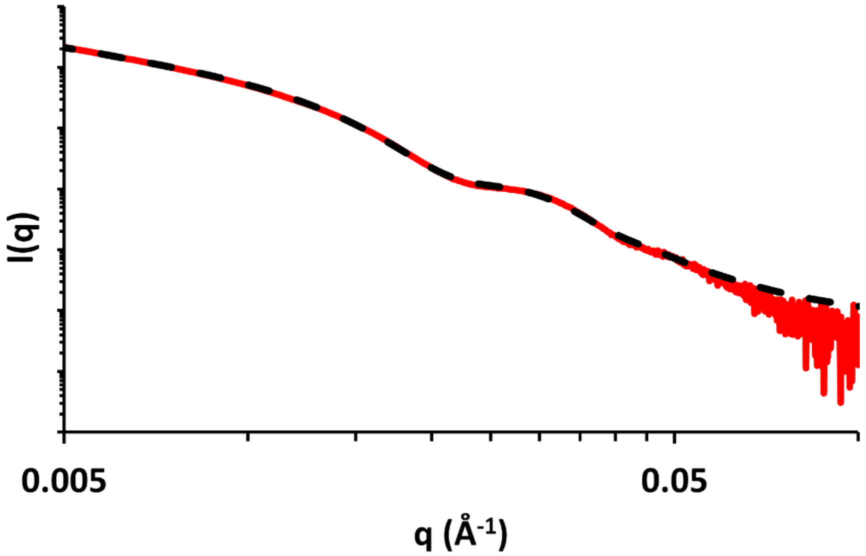

Figure S9. Background-subtracted SAXS data obtained for 1.0% w/w $PHEMA_{30}$-$PBzMA_{196}$ in [EMIM][DCA] at 25 °C. Dashed lines represent the model fit obtained using a combination of the spherical micelle model and worm-like micelle model.

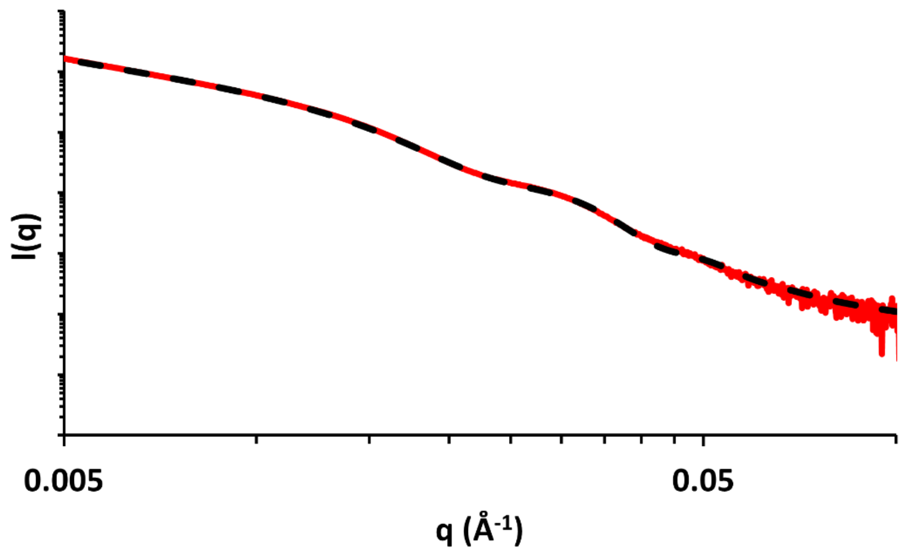

Figure S10. Background-subtracted SAXS data obtained for 1.0% w/w $PHEMA_{30}$-$PBzMA_{201}$ in [EMIM][DCA] at 25 °C. Dashed lines represent the model fit obtained using a combination of the spherical micelle model and worm-like micelle model.

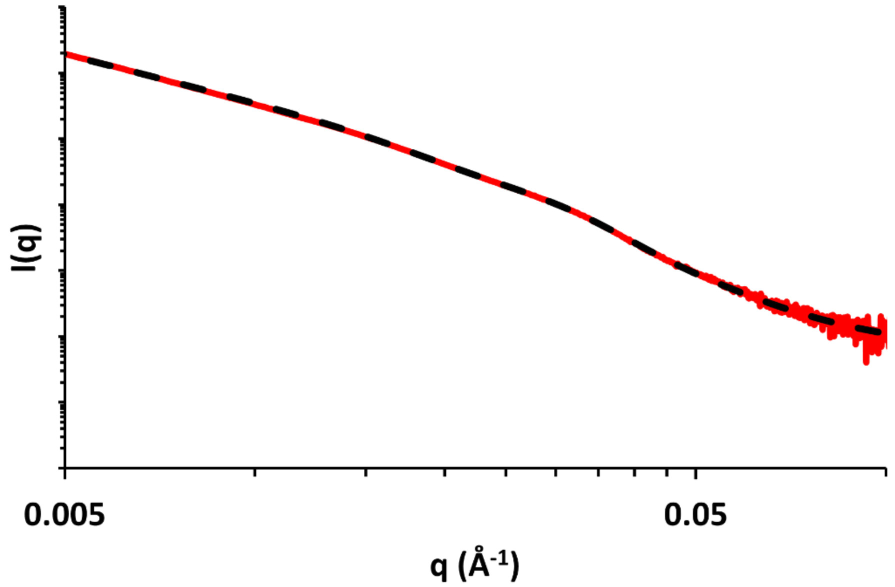

Figure S11. Background-subtracted SAXS data obtained for 1.0% w/w $PHEMA_{30}$-$PBzMA_{216}$ in [EMIM][DCA] at 25 °C. Dashed lines represent the model fit obtained using a combination of the spherical micelle model and worm-like micelle model.

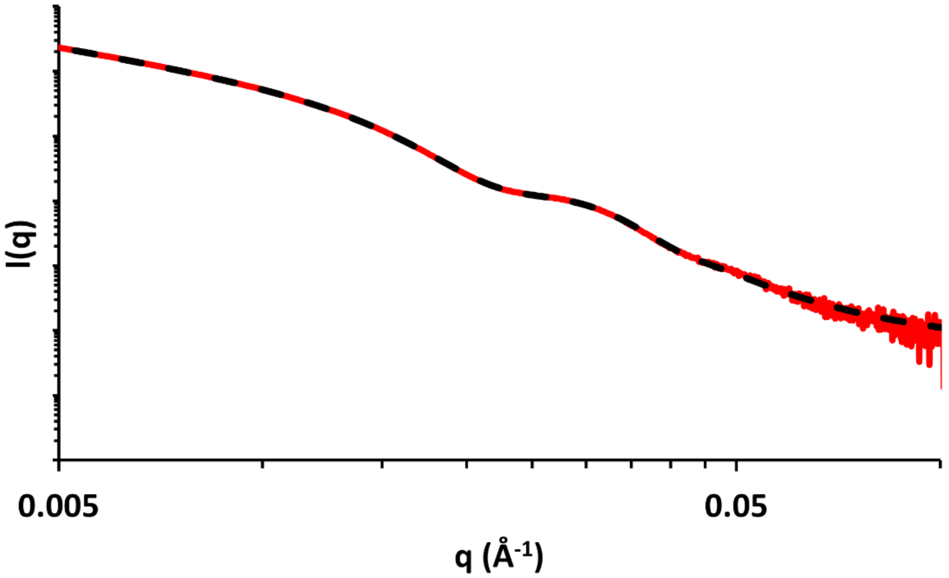

Figure S12. Background-subtracted SAXS data obtained for 1.0% w/w $PHEMA_{30}$-$PBzMA_{228}$ in [EMIM][DCA] at 25 °C. Dashed lines represent the model fit obtained using a combination of the spherical micelle model and worm-like micelle model.

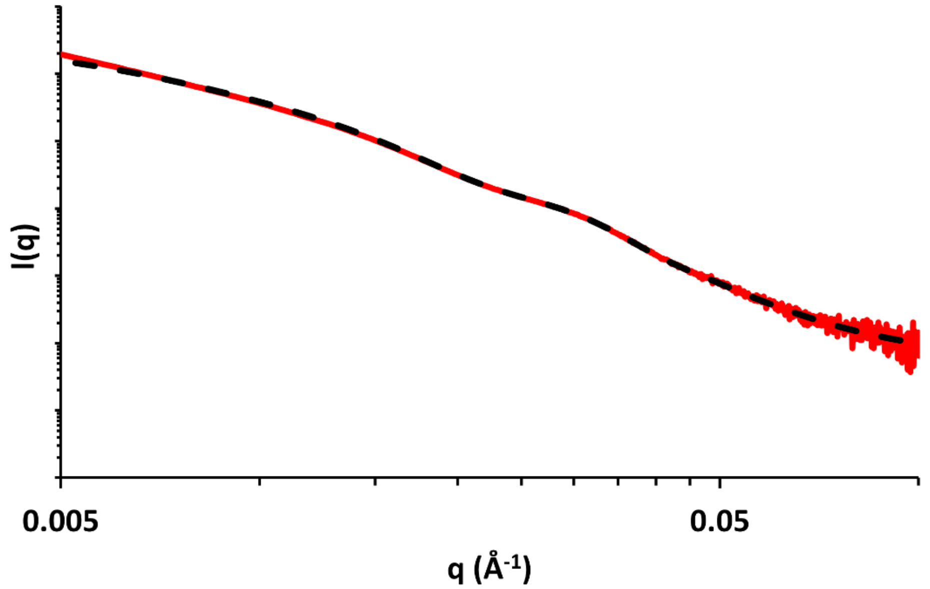

Figure S13. Background-subtracted SAXS data obtained for 1.0% w/w $PHEMA_{30}$-$PBzMA_{233}$ in [EMIM][DCA] at 25 °C. Dashed lines represent the model fit obtained using a combination of the spherical micelle model and worm-like micelle model.

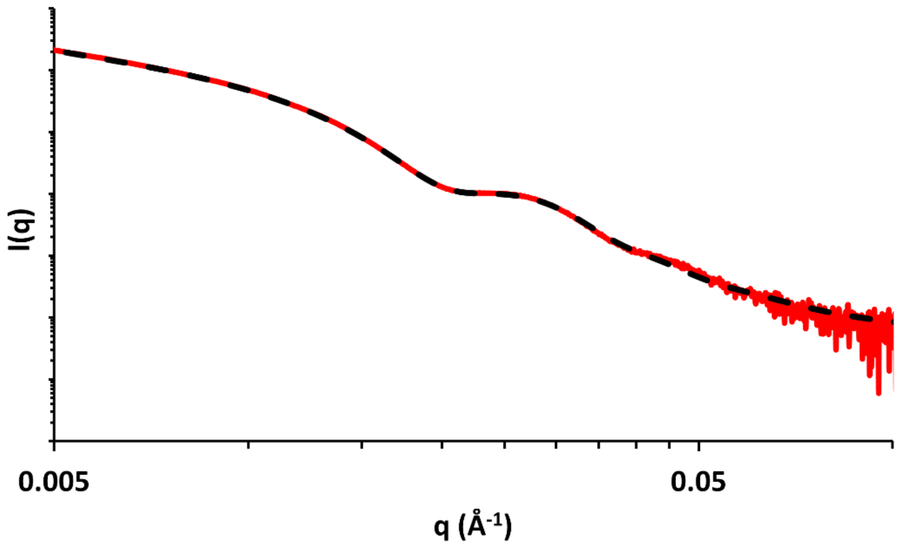

Figure S14. Background-subtracted SAXS data obtained for 1.0% w/w $PHEMA_{30}$-$PBzMA_{240}$ in [EMIM][DCA] at 25 °C. Dashed lines represent the model fit obtained using a combination of the spherical micelle model and worm-like micelle model.

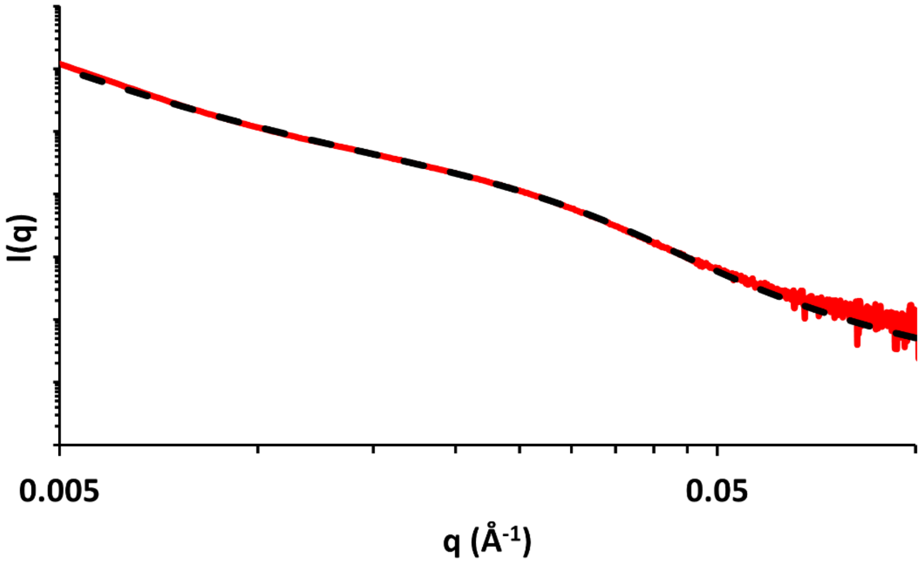

Figure S15. Background-subtracted SAXS data obtained for 1.0% w/w $PHEMA_{30}$-$PBzMA_{250}$ in [EMIM][DCA] at 25 °C. Dashed lines represent the model fit obtained using the worm-like micelle model.

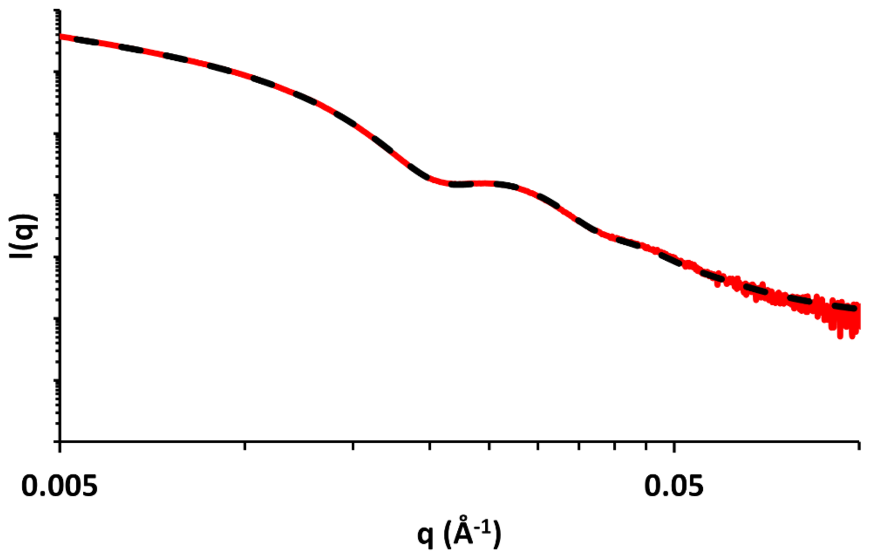

Figure S16. Background-subtracted SAXS data obtained for 1.0% w/w PHEMA$_{30}$-PBzMA$_{265}$ in [EMIM][DCA] at 25 °C. Dashed lines represent the model fit obtained using a combination of the spherical micelle model and worm-like micelle model.

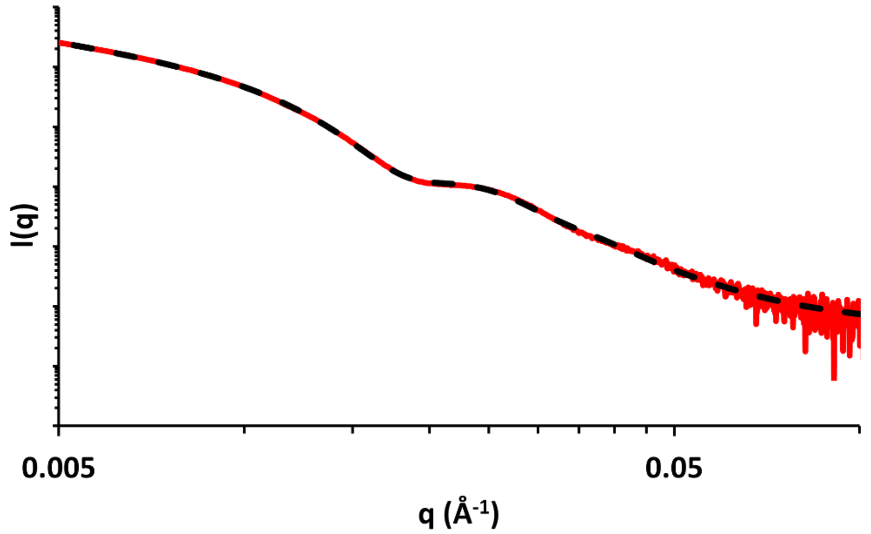

Figure S17. Background-subtracted SAXS data obtained for 1.0% w/w PHEMA$_{30}$-PBzMA$_{269}$ in [EMIM][DCA] at 25 °C. Dashed lines represent the model fit obtained a combination of the spherical micelle model and worm-like micelle model.

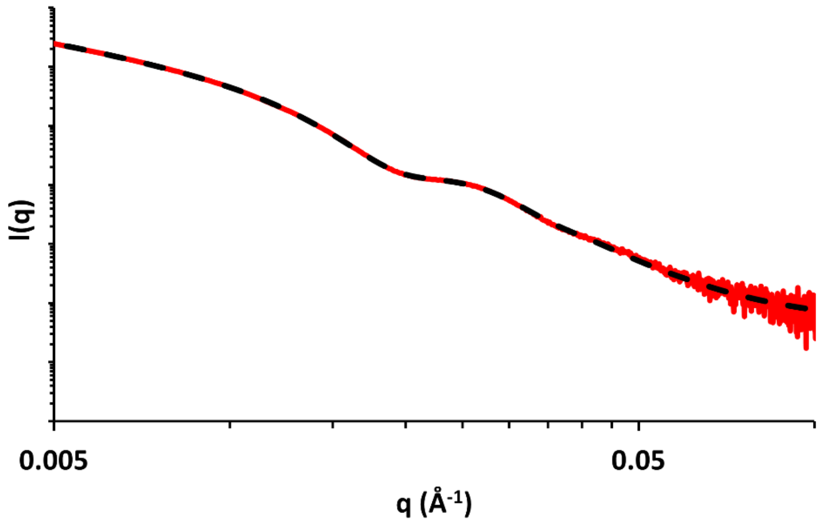

Figure S18. Background-subtracted SAXS data obtained for 1.0% w/w PHEMA$_{30}$-PBzMA$_{279}$ in [EMIM][DCA] at 25 °C. Dashed lines represent the model fit obtained using a combination of the spherical micelle model and worm-like micelle model.

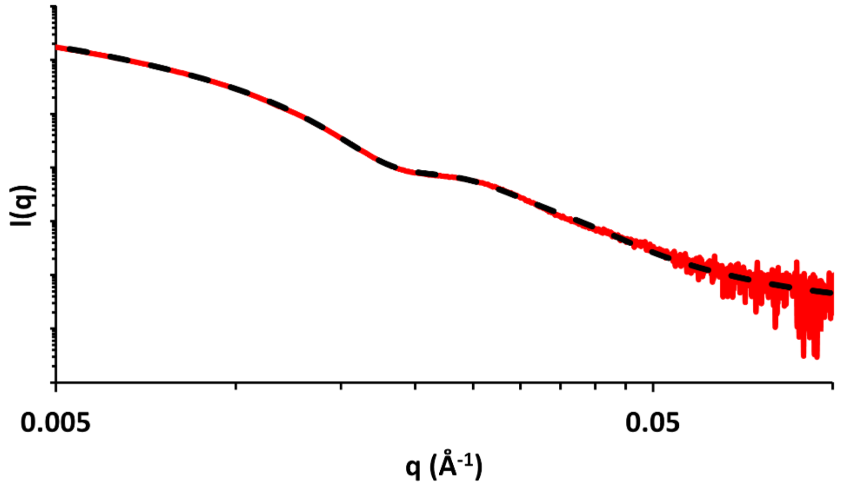

Figure S19. Background-subtracted SAXS data obtained for 1.0% w/w PHEMA$_{30}$-PBzMA$_{291}$ in [EMIM][DCA] at 25 °C. Dashed lines represent the model fit obtained using a combination of the spherical micelle model and worm-like micelle model.

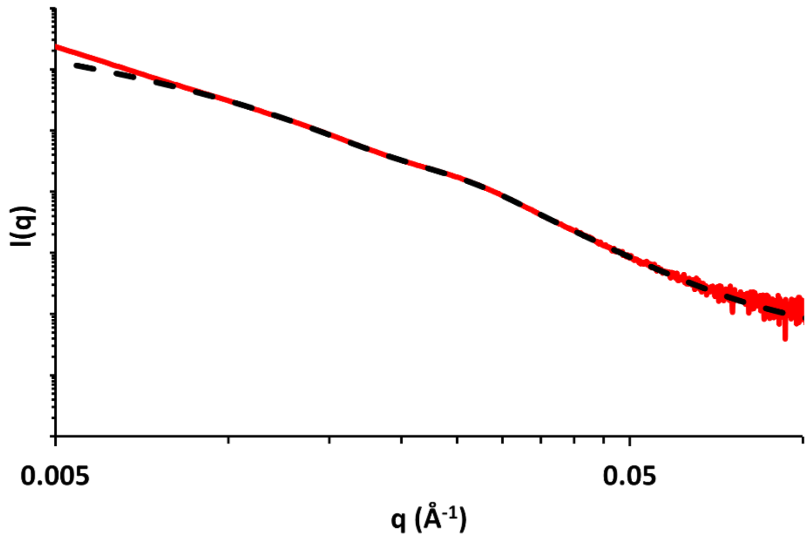

Figure S20. Background-subtracted SAXS data obtained for 1.0% w/w PHEMA$_{30}$-PBzMA$_{301}$ in [EMIM][DCA] at 25 °C. Dashed lines represent the model fit obtained using a combination of the spherical micelle model and worm-like micelle model.

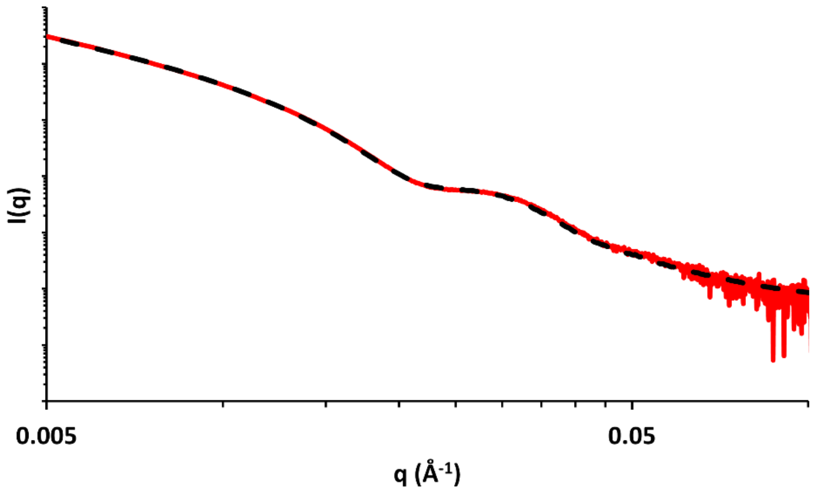

Figure S21. Background-subtracted SAXS data obtained for 1.0% w/w PHEMA$_{30}$-PBzMA$_{314}$ in [EMIM][DCA] at 25 °C. Dashed lines represent the model fit obtained using a combination of the worm-like micelle and vesicle models.

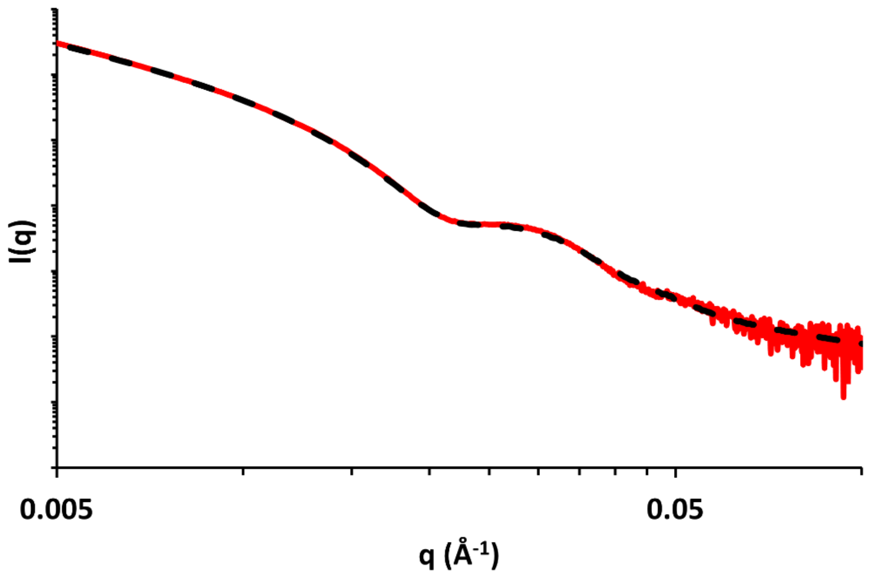

Figure S22. Background-subtracted SAXS data obtained for 1.0% w/w PHEMA$_{30}$-PBzMA$_{317}$ in [EMIM][DCA] at 25 °C. Dashed lines represent the model fit obtained using a combination of the worm-like micelle and vesicle models.

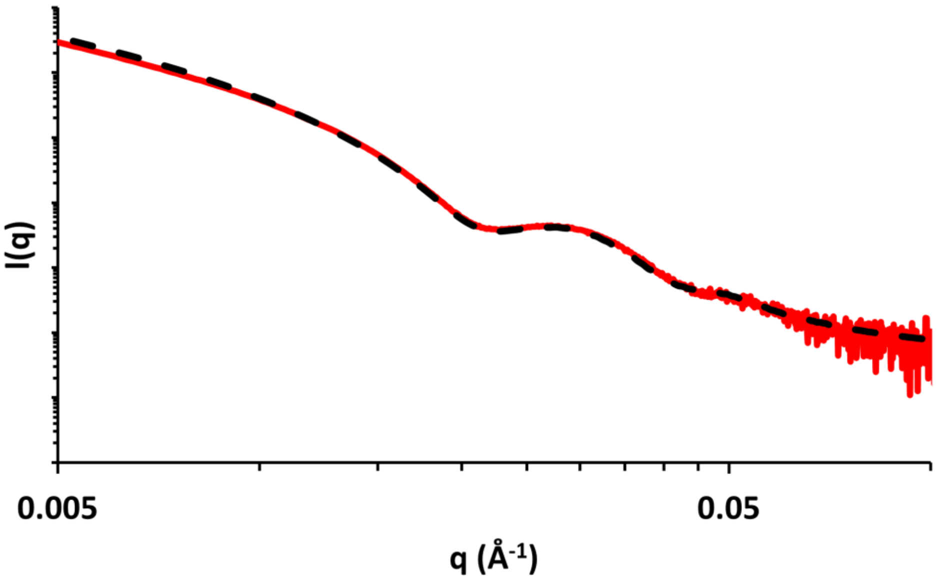

Figure S23. Background-subtracted SAXS data obtained for 1.0% w/w PHEMA$_{30}$-PBzMA$_{330}$ in [EMIM][DCA] at 25 °C. Dashed lines represent the model fit obtained using a combination of the worm-like micelle and vesicle models.

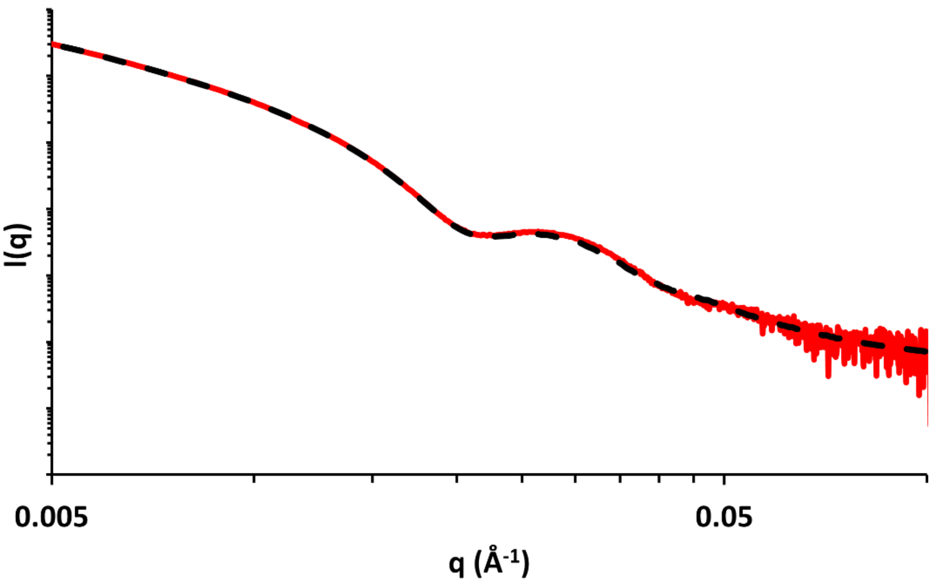

Figure S24. Background-subtracted SAXS data obtained for 1.0% w/w $PHEMA_{30}$-$PBzMA_{340}$ in [EMIM][DCA] at 25 °C. Dashed lines represent the model fit obtained using a combination of the worm-like micelle and vesicle models.

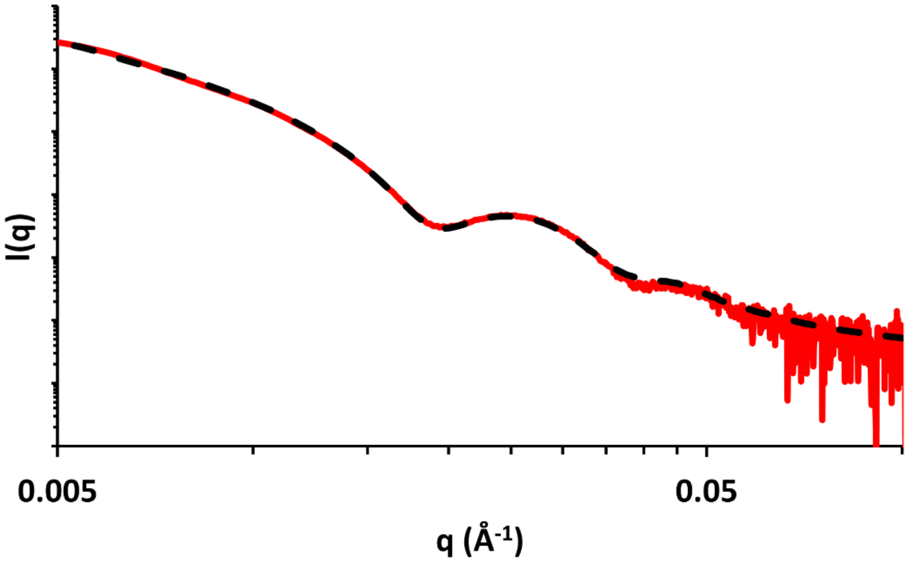

Figure S25. Background-subtracted SAXS data obtained for 1.0% w/w $PHEMA_{30}$-$PBzMA_{396}$ in [EMIM][DCA] at 25 °C. Dashed lines represent the model fit obtained using the vesicle model.

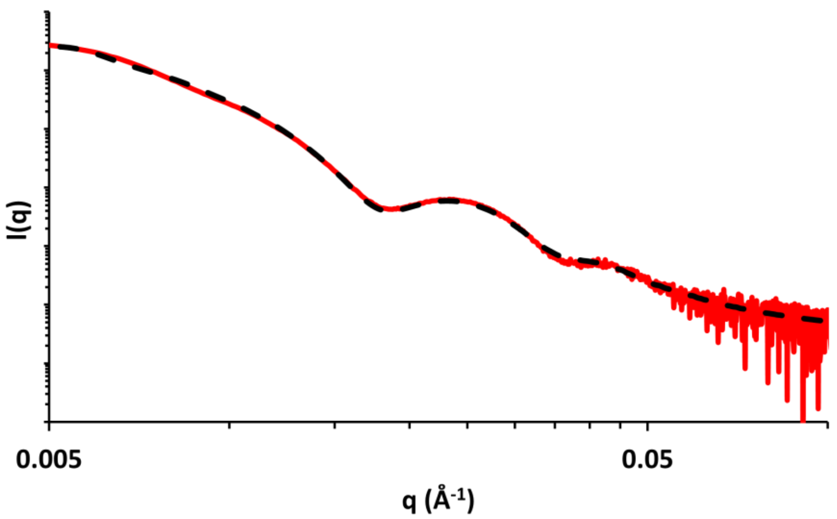

Figure S26. Background-subtracted SAXS data obtained for 1.0% w/w $PHEMA_{30}$-$PBzMA_{446}$ in [EMIM][DCA] at 25 °C. Dashed lines represent the model fit obtained using a vesicle model.

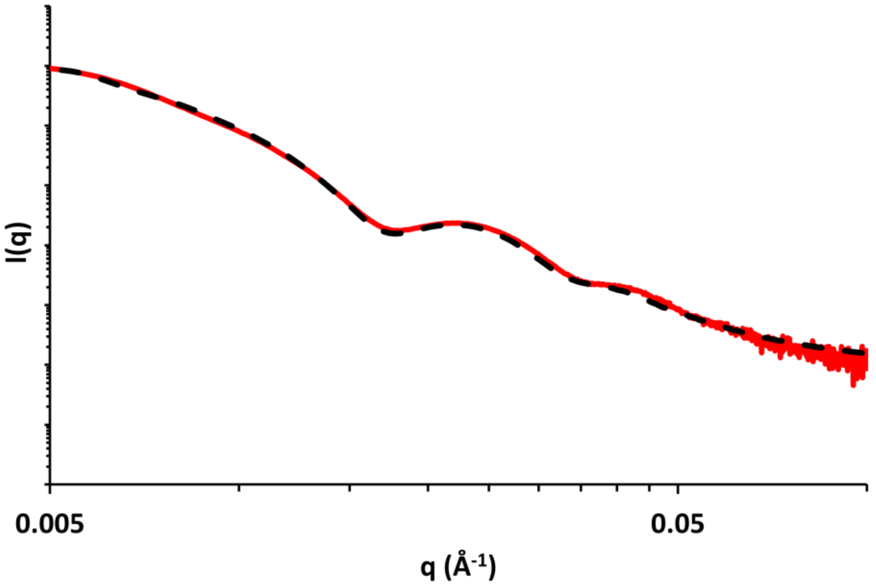

Figure S27. Background-subtracted SAXS data obtained for 1.0% w/w $PHEMA_{30}$-$PBzMA_{490}$ in [EMIM][DCA] at 25 °C. Dashed lines represent the model fit obtained using a vesicle model.

Additional oscillatory rheology data

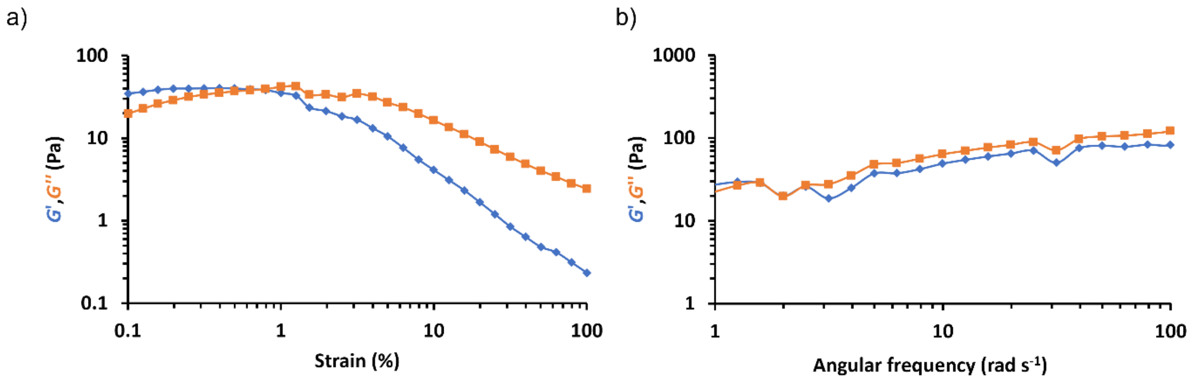

Figure S28. Oscillatory rheology data obtained for 15% w/w PHEMA$_{30}$-PBzMA$_{49}$ in [EMIM][DCA] at 25 °C. a) Strain sweep at a fixed angular frequency of 6.28 rad s^{-1} and b) frequency sweep at fixed a strain of 1.0%.

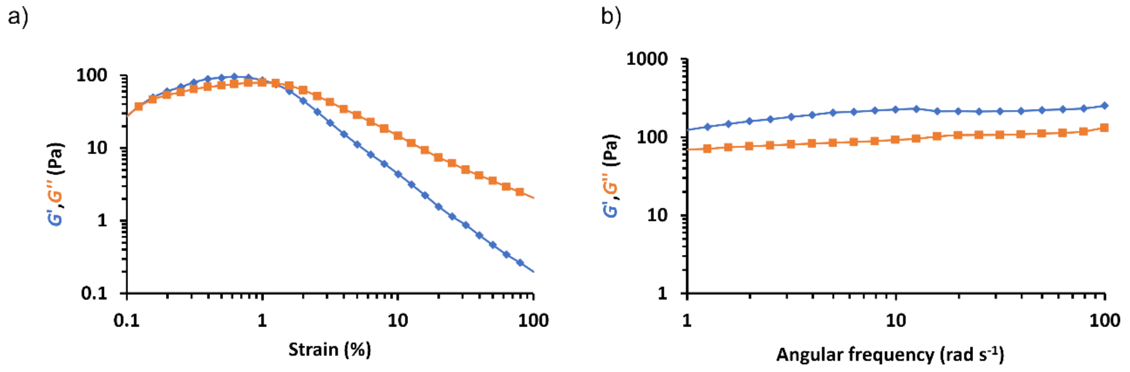

Figure S29. Oscillatory rheology data obtained for 15% w/w PHEMA$_{30}$-PBzMA$_{98}$ in [EMIM][DCA] at 25 °C. a) Strain sweep at a fixed angular frequency of 6.28 rad s^{-1} and b) frequency sweep at fixed a strain of 1.0%.

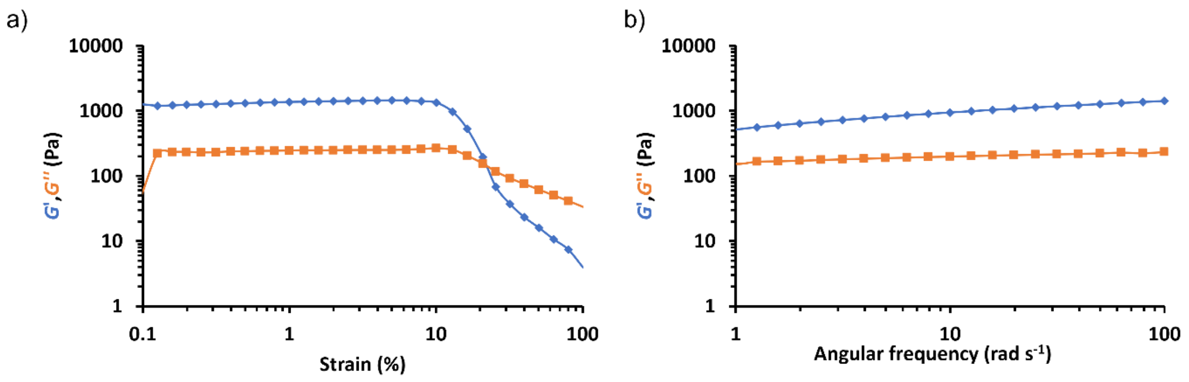

Figure S30. Oscillatory rheology data obtained for 15% w/w $PHEMA_{30}$-$PBzMA_{146}$ in [EMIM][DCA] at 25 °C. a) Strain sweep at a fixed angular frequency of 6.28 rad s^{-1} and b) frequency sweep at fixed a strain of 1.0%.

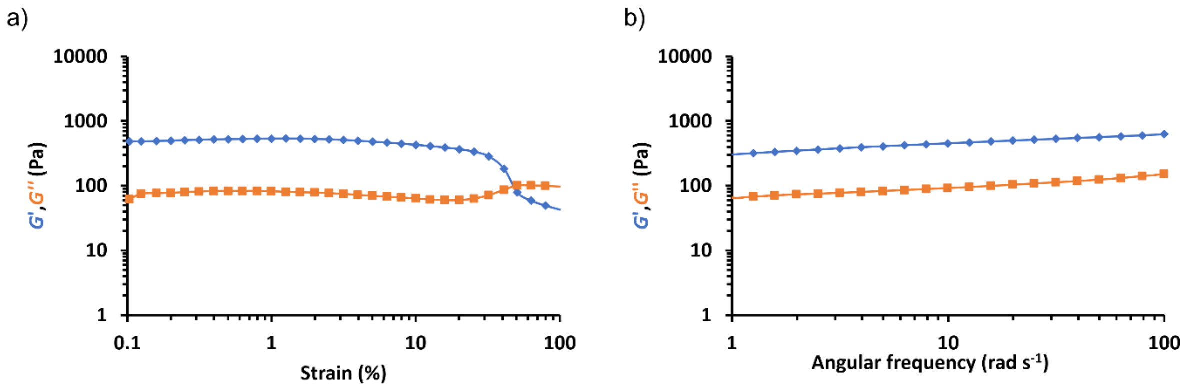

Figure S31. Oscillatory rheology data obtained for 15% w/w $PHEMA_{30}$-$PBzMA_{196}$ in [EMIM][DCA] at 25 °C. a) Strain sweep at a fixed angular frequency of 6.28 rad s^{-1} and b) frequency sweep at fixed a strain of 1.0%.

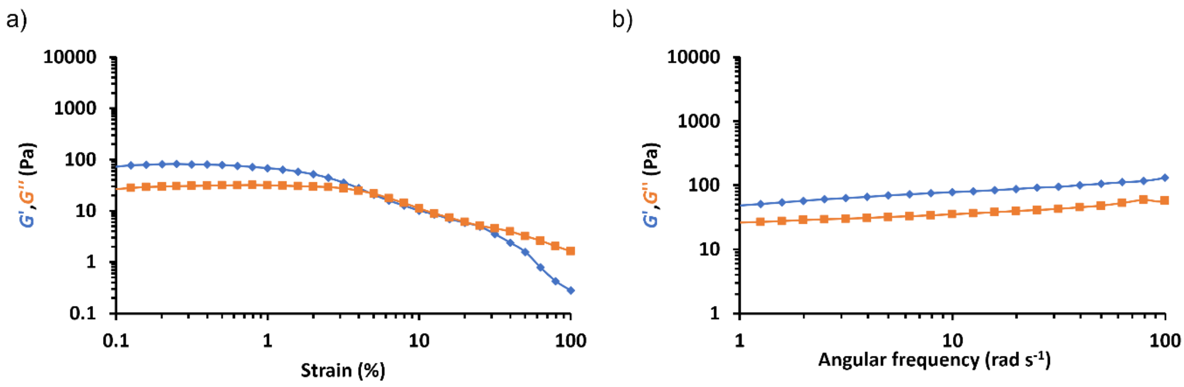

Figure S32. Oscillatory rheology data obtained for 15% w/w $PHEMA_{30}$-$PBzMA_{201}$ in [EMIM][DCA] at 25 °C. a) Strain sweep at a fixed angular frequency of 6.28 rad s^{-1} and b) frequency sweep at fixed a strain of 1.0%.

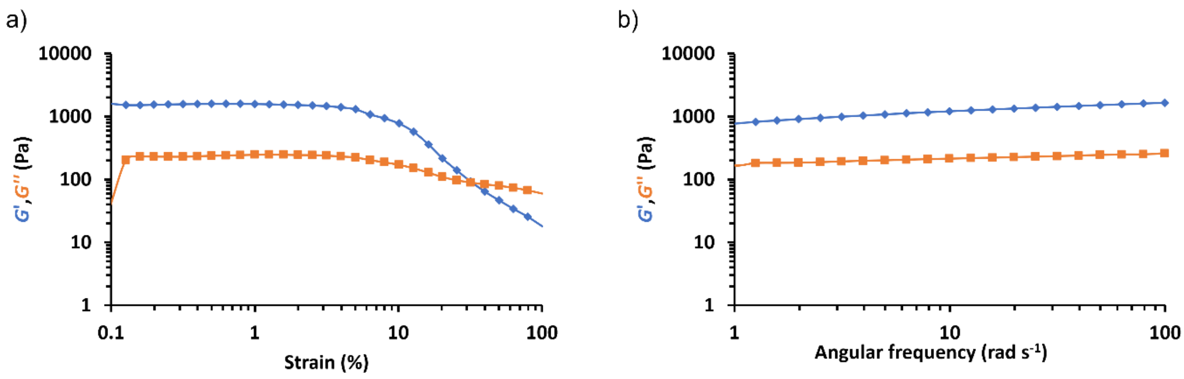

Figure S33. Oscillatory rheology data obtained for 15% w/w $PHEMA_{30}$-$PBzMA_{216}$ in [EMIM][DCA] at 25 °C. a) Strain sweep at a fixed angular frequency of 6.28 rad s^{-1} and b) frequency sweep at fixed a strain of 1.0%.

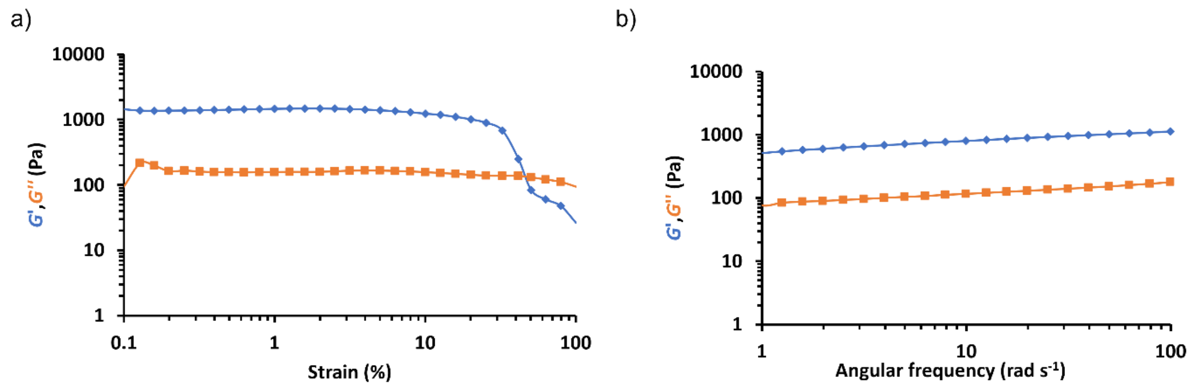

Figure S34. Oscillatory rheology data obtained for 15% w/w PHEMA$_{30}$-PBzMA$_{228}$ in [EMIM][DCA] at 25 °C. a) Strain sweep at a fixed angular frequency of 6.28 rad s^{-1} and b) frequency sweep at fixed a strain of 1.0%.

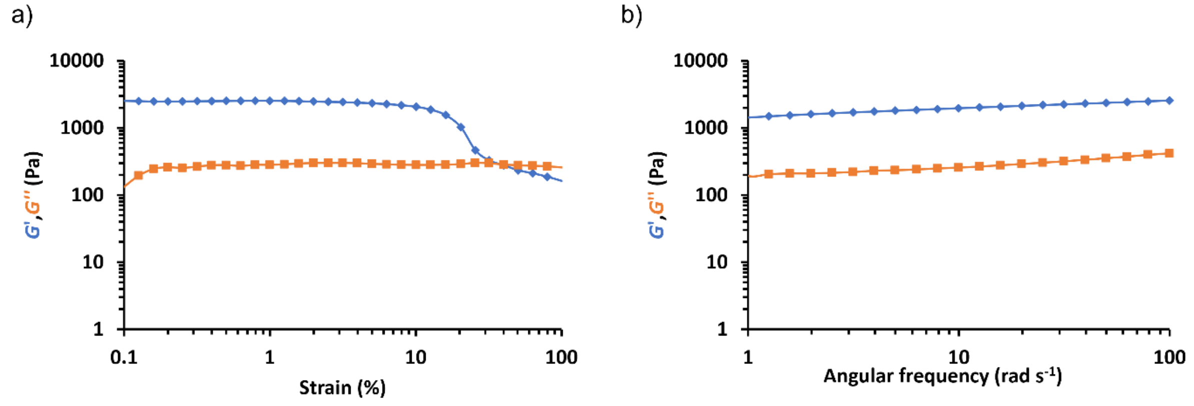

Figure S35. Oscillatory rheology data obtained for 15% w/w PHEMA$_{30}$-PBzMA$_{233}$ in [EMIM][DCA] at 25 °C. a) Strain sweep at a fixed angular frequency of 6.28 rad s^{-1} and b) frequency sweep at fixed a strain of 1.0%.

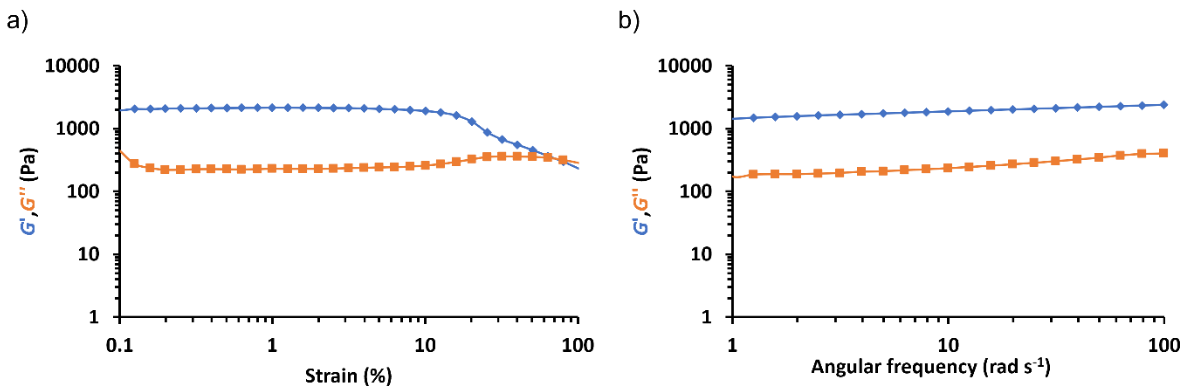

Figure S36. Oscillatory rheology data obtained for 15% w/w PHEMA$_{30}$-PBzMA$_{243}$ in [EMIM][DCA] at 25 °C. a) Strain sweep at a fixed angular frequency of 6.28 rad s^{-1} and b) frequency sweep at fixed a strain of 1.0%.

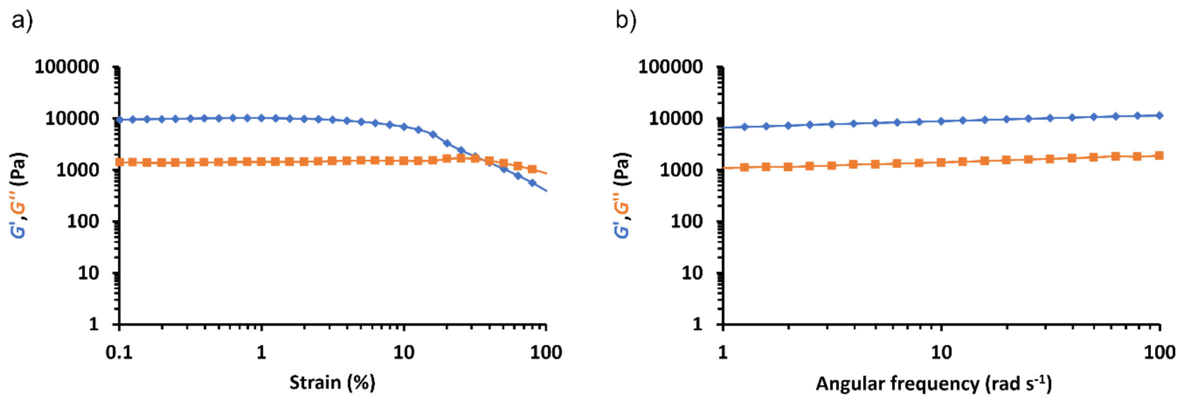

Figure S37. Oscillatory rheology data obtained for 15% w/w PHEMA$_{30}$-PBzMA$_{250}$ in [EMIM][DCA] at 25 °C. a) Strain sweep at a fixed angular frequency of 6.28 rad s^{-1} and b) frequency sweep at fixed a strain of 1.0%.

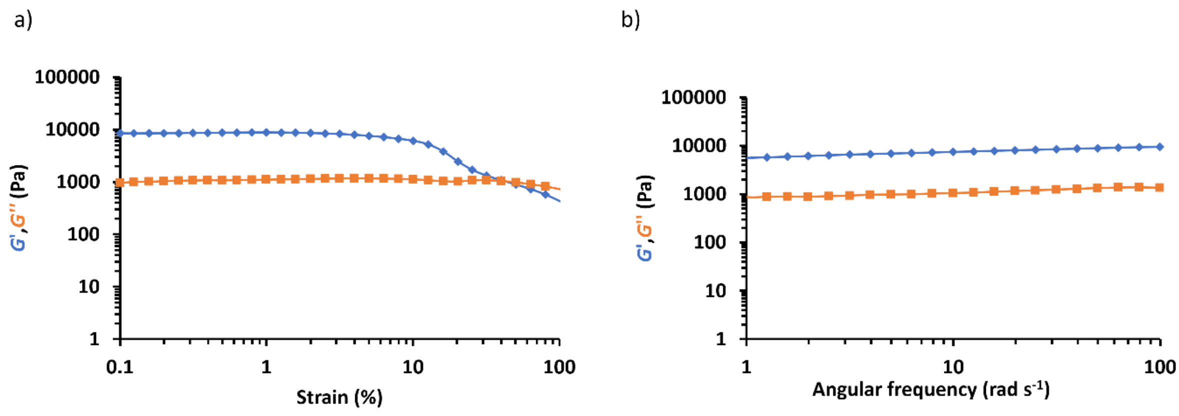

Figure S38. Oscillatory rheology data obtained for 15% w/w $PHEMA_{30}$-$PBzMA_{265}$ in [EMIM][DCA] at 25 °C. a) Strain sweep at a fixed angular frequency of 6.28 rad s^{-1} and b) frequency sweep at fixed a strain of 1.0%.

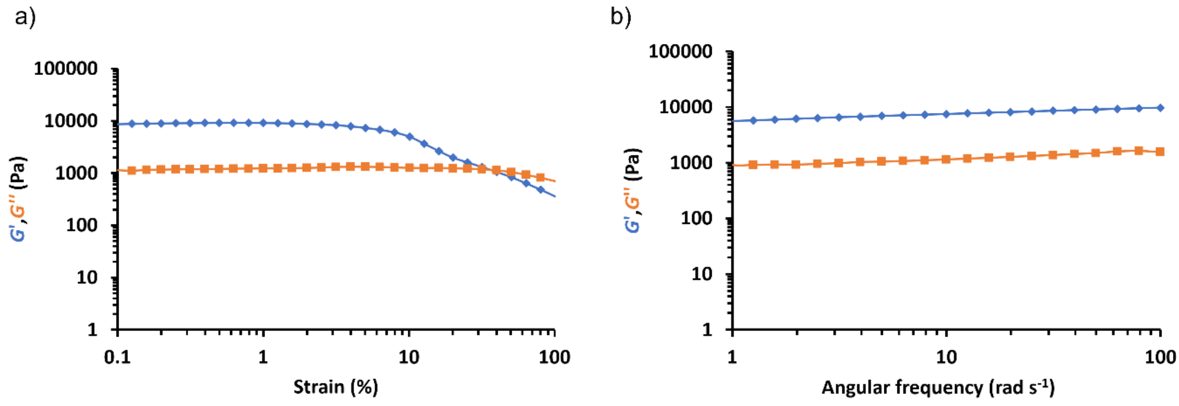

Figure S39. Oscillatory rheology data obtained for 15% w/w $PHEMA_{30}$-$PBzMA_{269}$ in [EMIM][DCA] at 25 °C. a) Strain sweep at a fixed angular frequency of 6.28 rad s^{-1} and b) frequency sweep at fixed a strain of 1.0%.

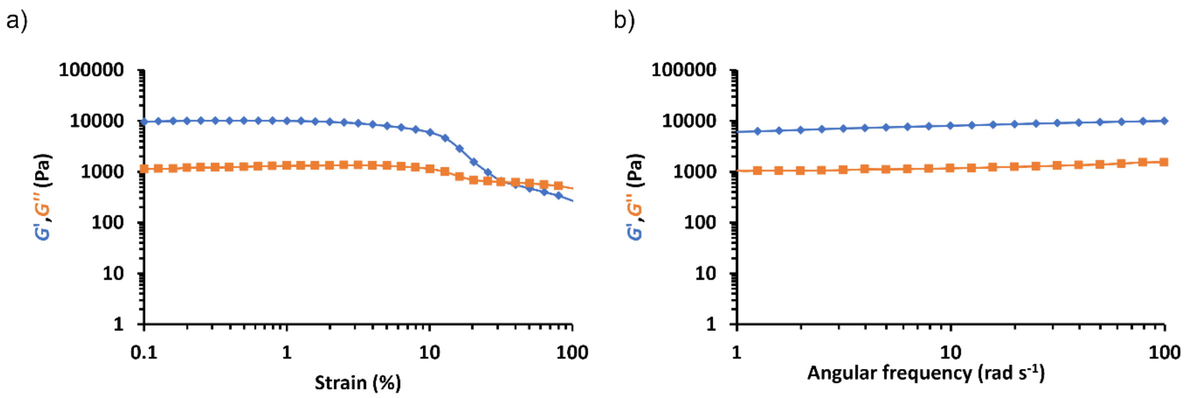

Figure S40. Oscillatory rheology data obtained for 15% w/w $PHEMA_{30}$-$PBzMA_{279}$ in [EMIM][DCA] at 25 °C. a) Strain sweep at a fixed angular frequency of 6.28 rad s^{-1} and b) frequency sweep at fixed a strain of 1.0%.

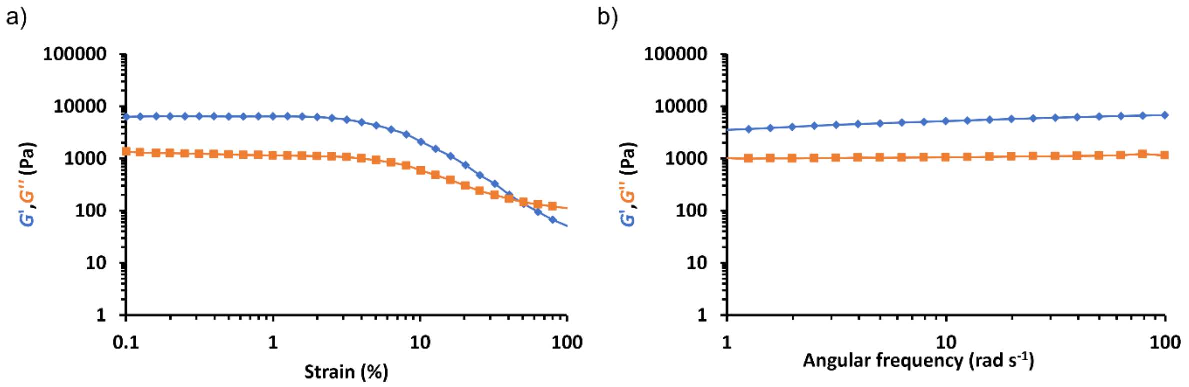

Figure S41. Oscillatory rheology data obtained for 15% w/w $PHEMA_{30}$-$PBzMA_{301}$ in [EMIM][DCA] at 25 °C. a) Strain sweep at a fixed angular frequency of 6.28 rad s^{-1} and b) frequency sweep at fixed a strain of 1.0%.

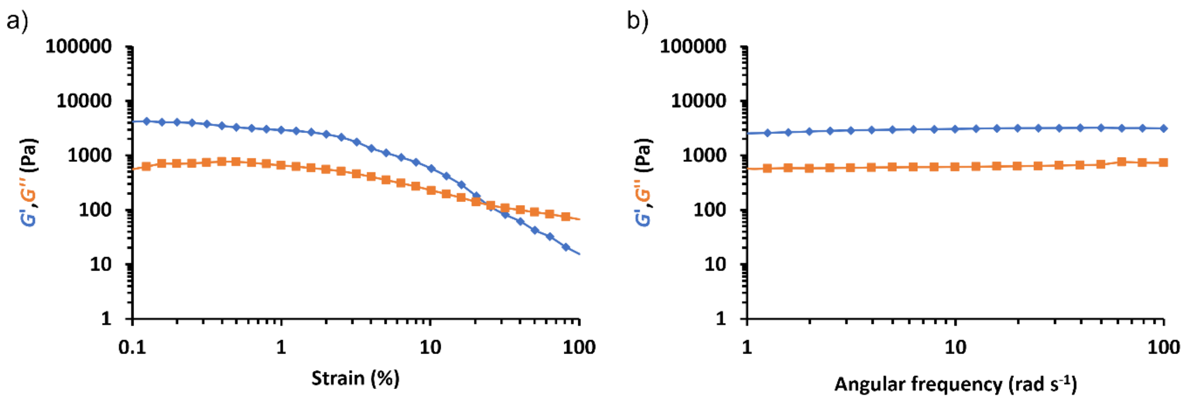

Figure S42. Oscillatory rheology data obtained for 15% w/w $PHEMA_{30}$-$PBzMA_{314}$ in [EMIM][DCA] at 25 °C. a) Strain sweep at a fixed angular frequency of 6.28 rad s^{-1} and b) frequency sweep at fixed a strain of 1.0%.

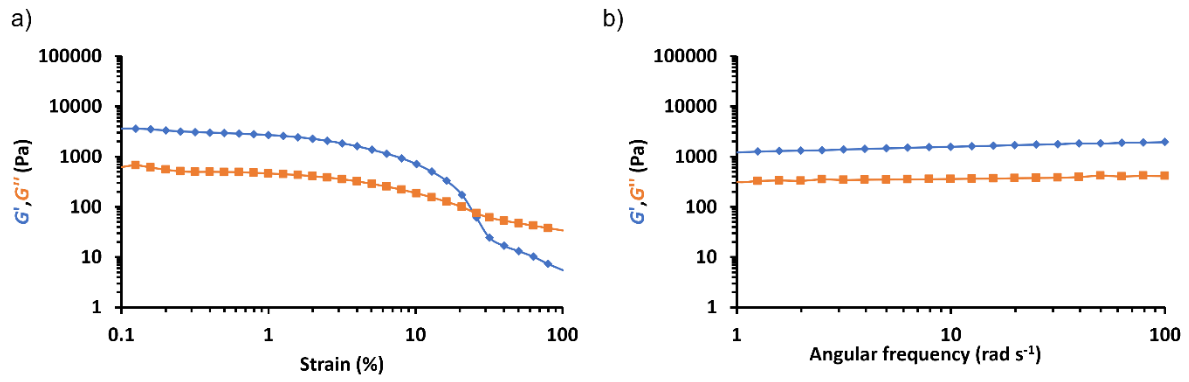

Figure S43. Oscillatory rheology data obtained for 15% w/w $PHEMA_{30}$-$PBzMA_{317}$ in [EMIM][DCA] at 25 °C. a) Strain sweep at a fixed angular frequency of 6.28 rad s^{-1} and b) frequency sweep at fixed a strain of 1.0%.

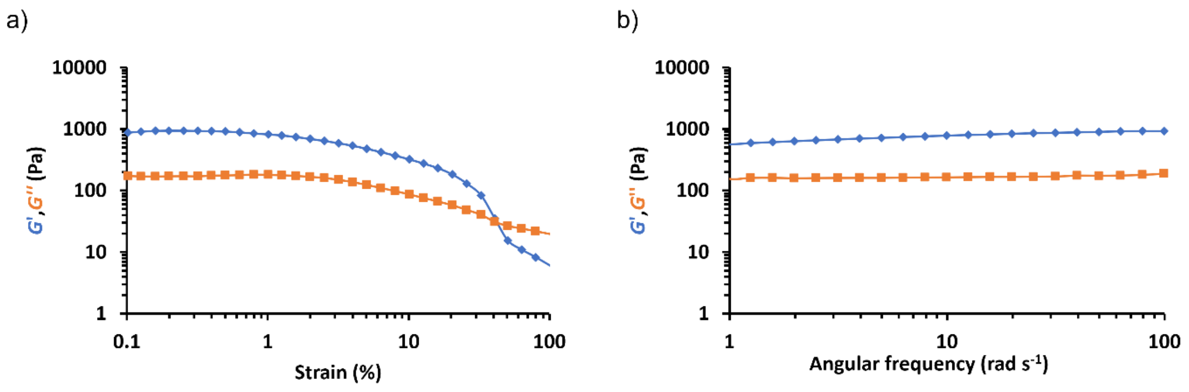

Figure S44. Oscillatory rheology data obtained for 15% w/w PHEMA$_{30}$-PBzMA$_{330}$ in [EMIM][DCA] at 25 °C. a) Strain sweep at a fixed angular frequency of 6.28 rad s^{-1} and b) frequency sweep at fixed a strain of 1.0%.

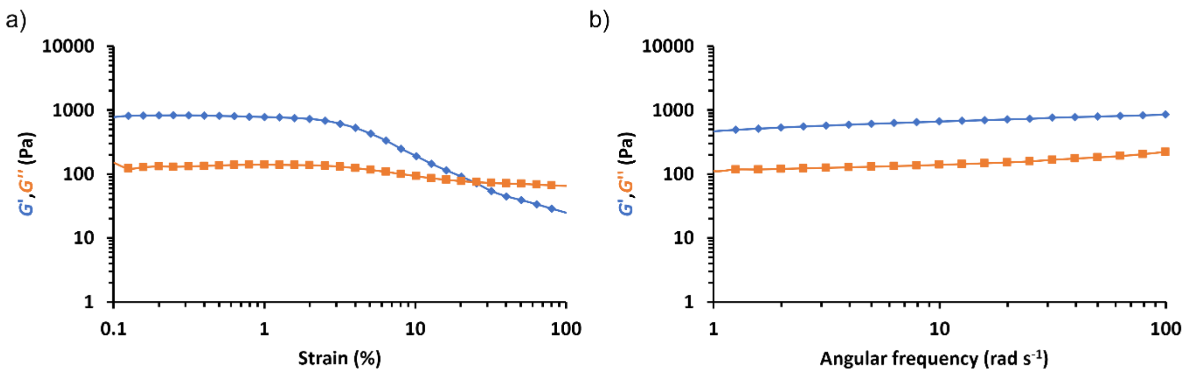

Figure S45. Oscillatory rheology data obtained for 15% w/w PHEMA$_{30}$-PBzMA$_{340}$ in [EMIM][DCA] at 25 °C. a) Strain sweep at a fixed angular frequency of 6.28 rad s^{-1} and b) frequency sweep at fixed a strain of 1.0%.

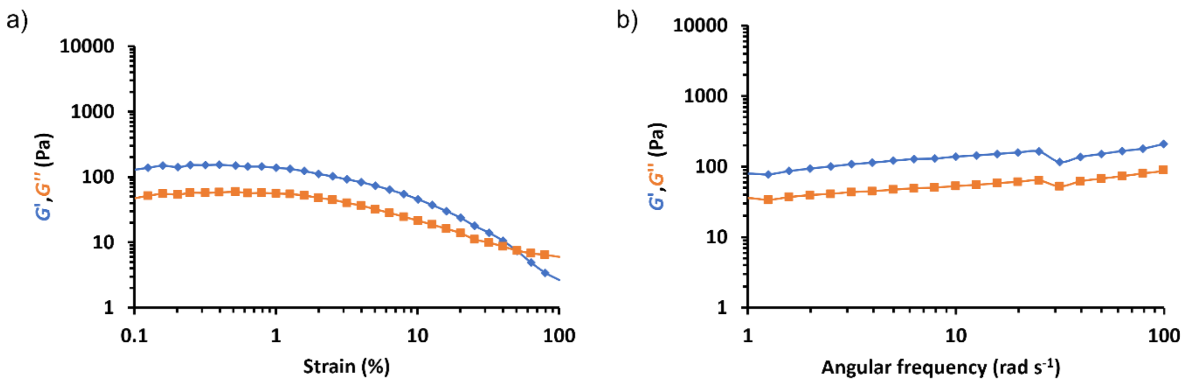

Figure S46. Oscillatory rheology data obtained for 15% w/w $PHEMA_{30}$-$PBzMA_{396}$ in [EMIM][DCA] at 25 °C. a) Strain sweep at a fixed angular frequency of 6.28 rad s^{-1} and b) frequency sweep at fixed a strain of 1.0%.

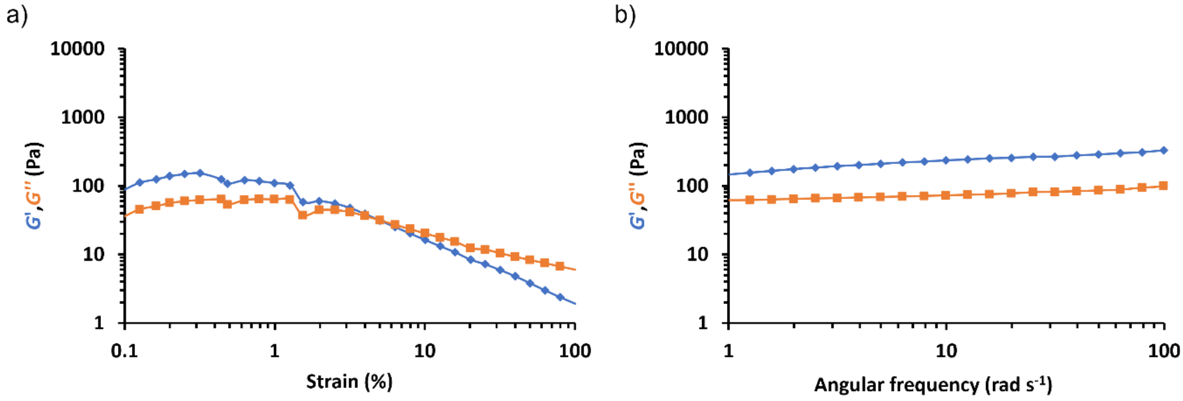

Figure S47. Oscillatory rheology data obtained for 15% w/w $PHEMA_{30}$-$PBzMA_{446}$ in [EMIM][DCA] at 25 °C. a) Strain sweep at a fixed angular frequency of 6.28 rad s^{-1} and b) frequency sweep at fixed a strain of 1.0%.

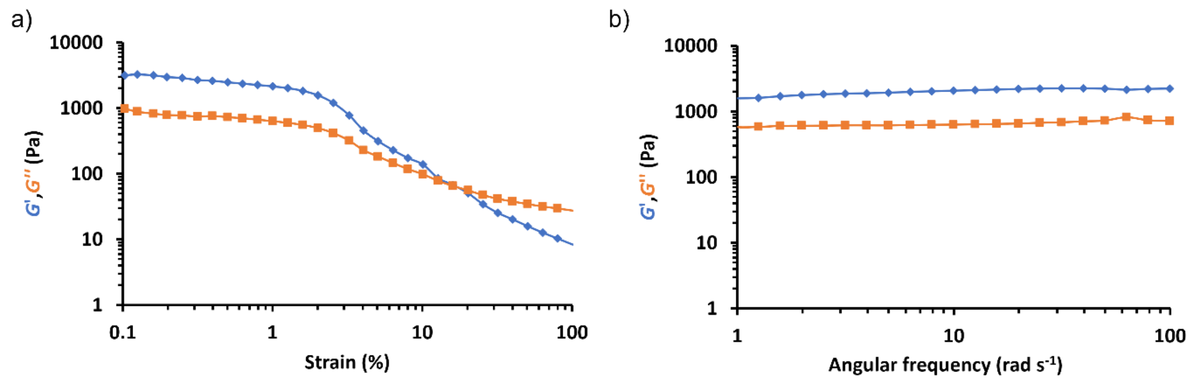

Figure S48. Oscillatory rheology data obtained for 15% w/w PHEMA$_{30}$-PBzMA$_{490}$ in [EMIM][DCA] at 25 °C. a) Strain sweep at a fixed angular frequency of 6.28 rad s^{-1} and b) frequency sweep at fixed a strain of 1.0%.

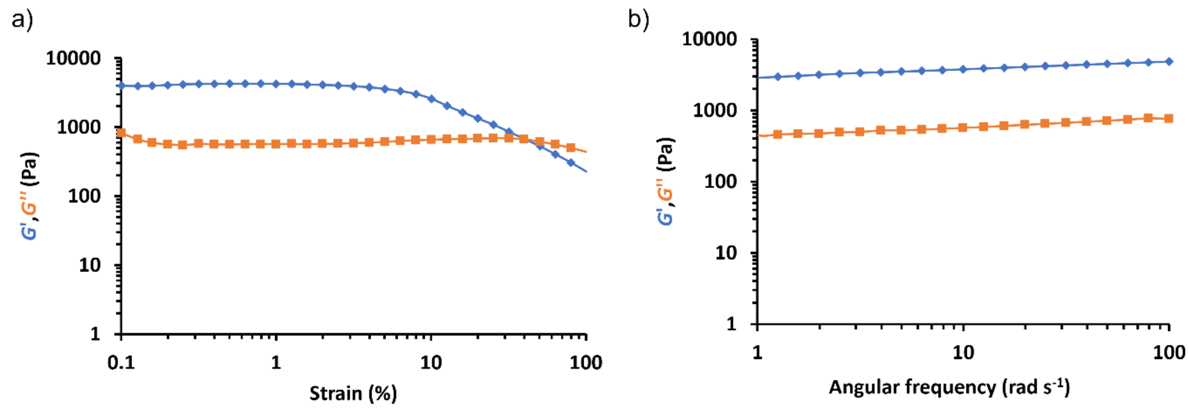

Figure S49. Oscillatory rheology data obtained for 10% w/w PHEMA$_{30}$-PBzMA$_{270}$ in [EMIM][DCA] at 25 °C. a) Strain sweep at a fixed angular frequency of 6.28 rad s^{-1} and b) frequency sweep at fixed a strain of 1.0%.

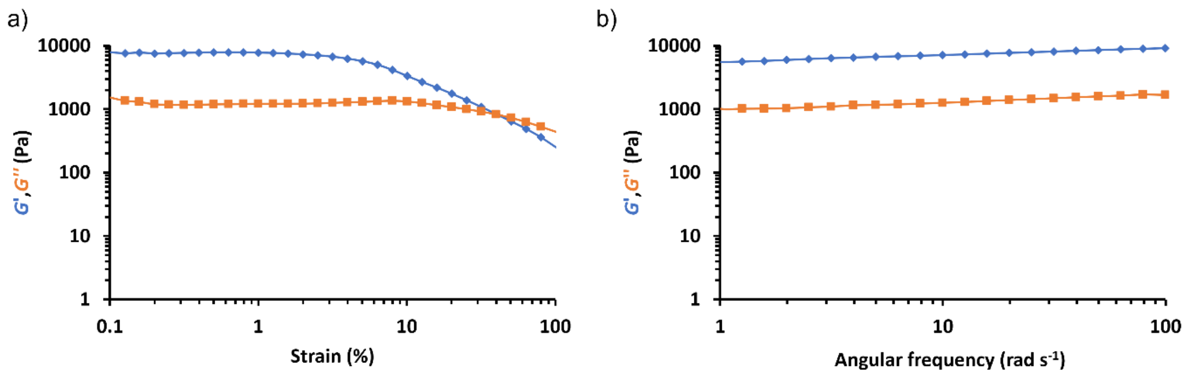

Figure S50. Oscillatory rheology data obtained for 9% w/w $PHEMA_{30}$-$PBzMA_{282}$ in [EMIM][DCA] at 25 °C. a) Strain sweep at a fixed angular frequency of 6.28 rad s^{-1} and b) frequency sweep at fixed a strain of 1.0%.

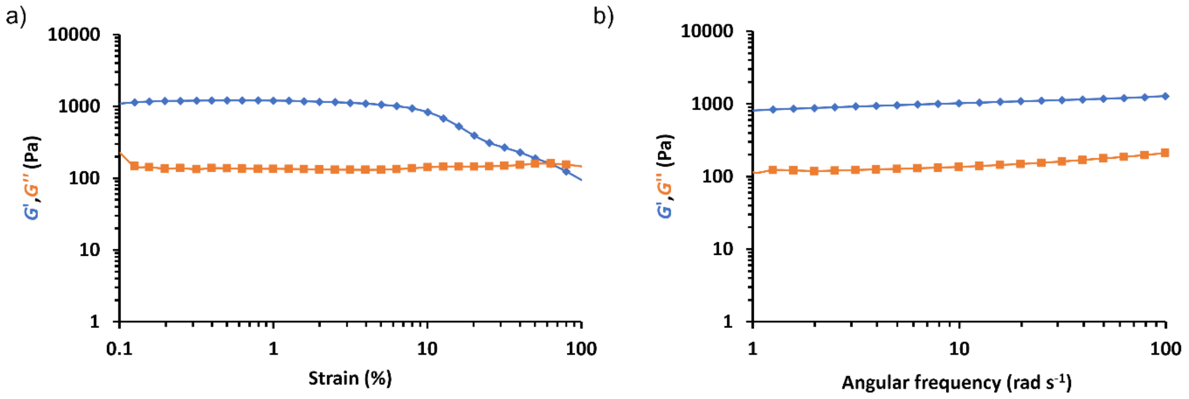

Figure S51. Oscillatory rheology data obtained for 8% w/w $PHEMA_{30}$-$PBzMA_{267}$ in [EMIM][DCA] at 25 °C. a) Strain sweep at a fixed angular frequency of 6.28 rad s^{-1} and b) frequency sweep at fixed a strain of 1.0%.

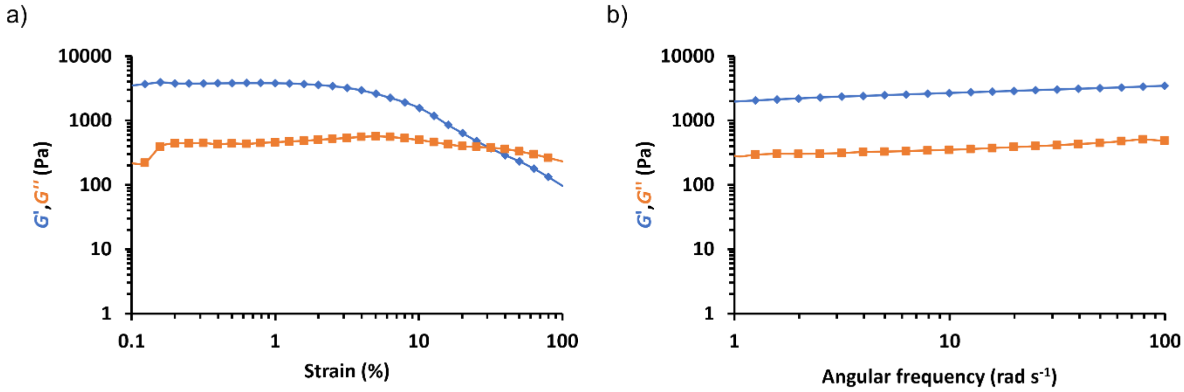

Figure S52. Oscillatory rheology data obtained for 7% w/w PHEMA$_{30}$-PBzMA$_{267}$ in [EMIM][DCA] at 25 °C. a) Strain sweep at a fixed angular frequency of 6.28 rad s^{-1} and b) frequency sweep at fixed a strain of 1.0%.

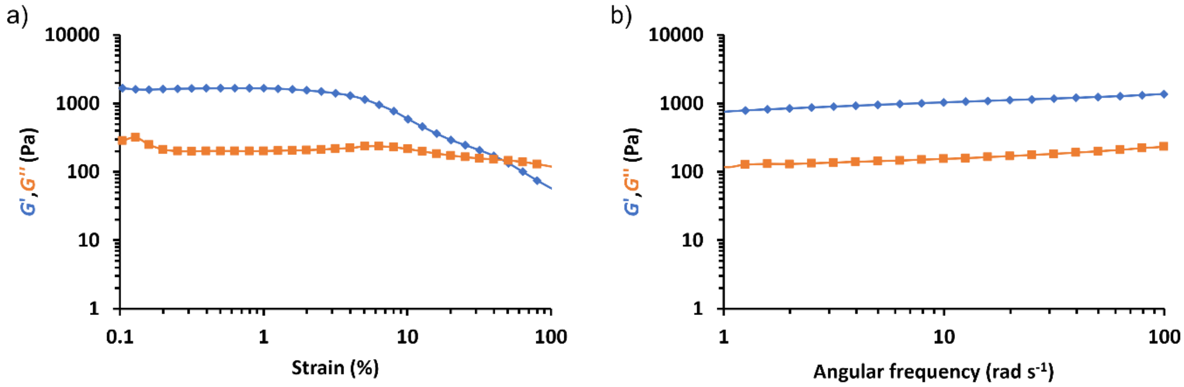

Figure S53. Oscillatory rheology data obtained for 6% w/w PHEMA$_{30}$-PBzMA$_{294}$ in [EMIM][DCA] at 25 °C. a) Strain sweep at a fixed angular frequency of 6.28 rad s^{-1} and b) frequency sweep at fixed a strain of 1.0%.

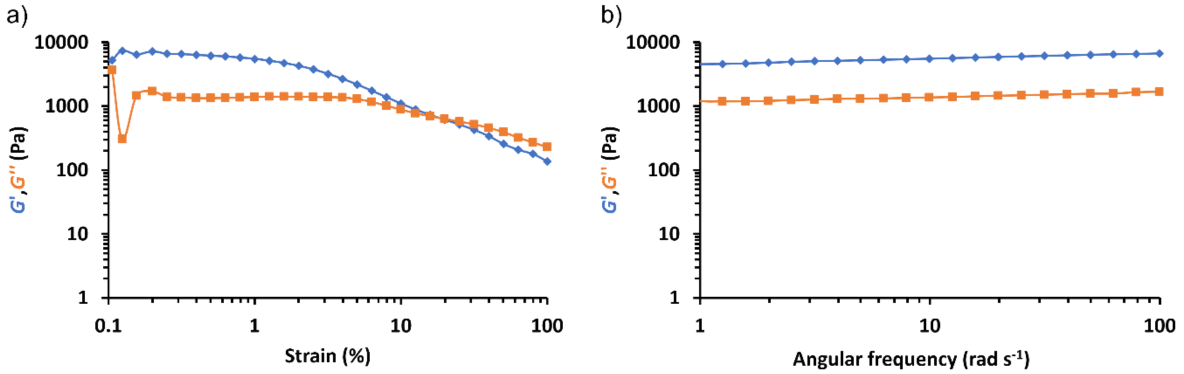

Figure S54. Oscillatory rheology data obtained for 5% w/w PHEMA$_{30}$-PBzMA$_{291}$ in [EMIM][DCA] at 25 °C. a) Strain sweep at a fixed angular frequency of 6.28 rad s^{-1} and b) frequency sweep at fixed a strain of 1.0%.

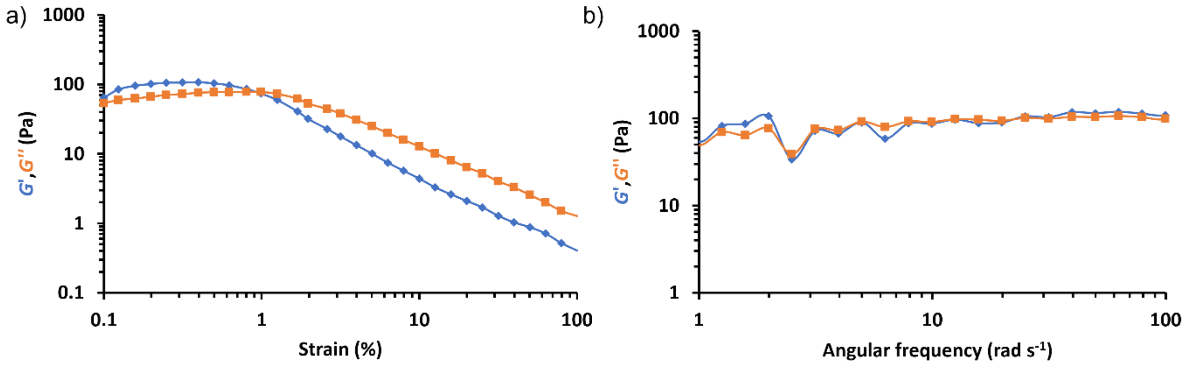

Figure S55. Oscillatory rheology data obtained for 4% w/w PHEMA$_{30}$-PBzMA$_{294}$ in [EMIM][DCA] at 25 °C. a) Strain sweep at a fixed angular frequency of 6.28 rad s^{-1} and b) frequency sweep at fixed a strain of 1.0%.

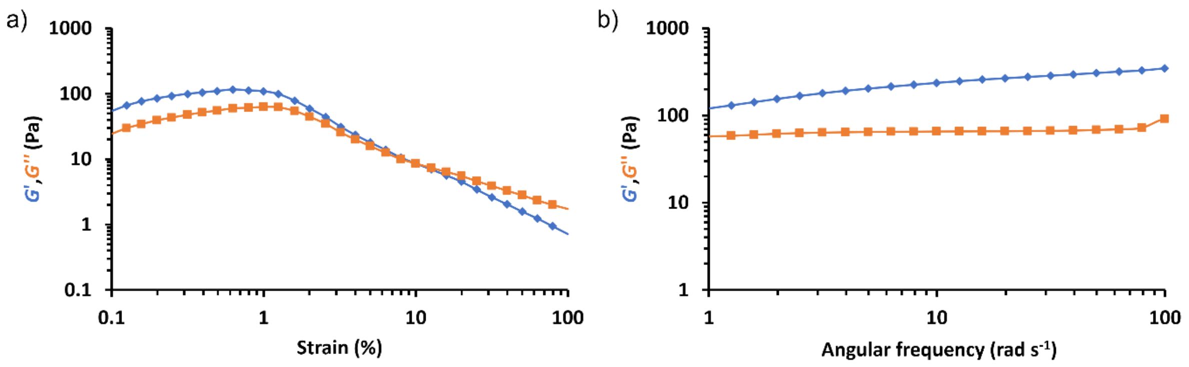

Figure S56. Oscillatory rheology data obtained for 3% w/w $PHEMA_{30}$-$PBzMA_{276}$ in [EMIM][DCA] at 25 °C. a) Strain sweep at a fixed angular frequency of 6.28 rad s^{-1} and b) frequency sweep at fixed a strain of 1.0%.

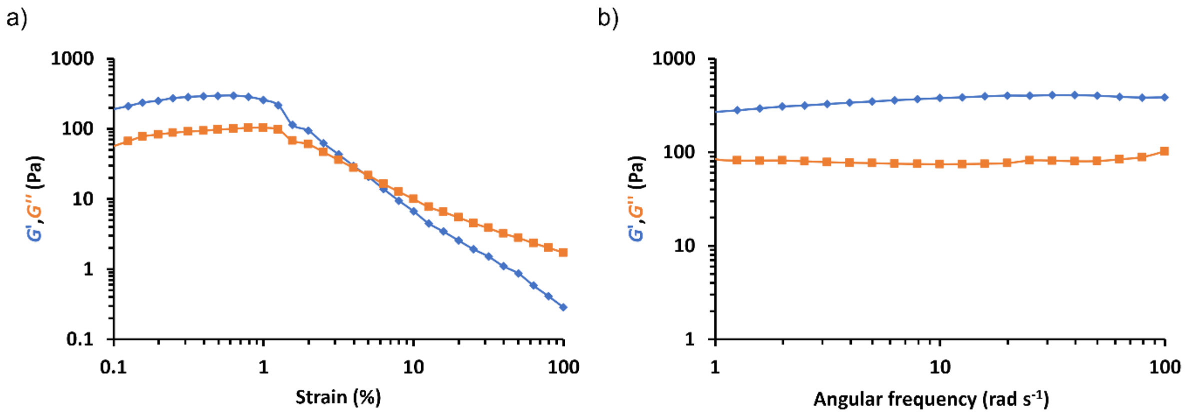

Figure S57. Oscillatory rheology data obtained for 2% w/w $PHEMA_{30}$-$PBzMA_{297}$ in [EMIM][DCA] at 25 °C. a) Strain sweep at a fixed angular frequency of 6.28 rad s^{-1} and b) frequency sweep at fixed a strain of 1.0%.

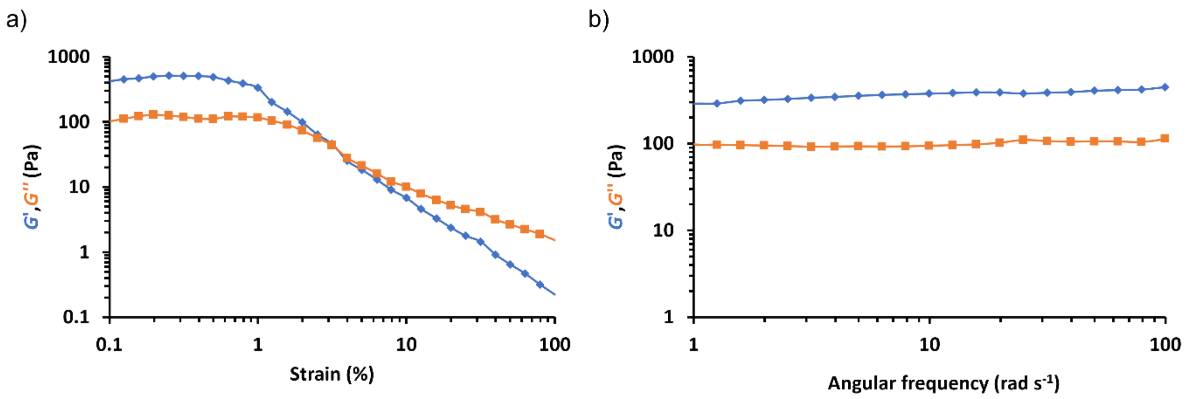

Figure S58. Oscillatory rheology data obtained for 1% w/w PHEMA$_{30}$-PBzMA$_{285}$ in [EMIM][DCA] at 25 °C. a) Strain sweep at a fixed angular frequency of 6.28 rad s^{-1} and b) frequency sweep at fixed a strain of 1.0%.

Thermogravimetric analysis

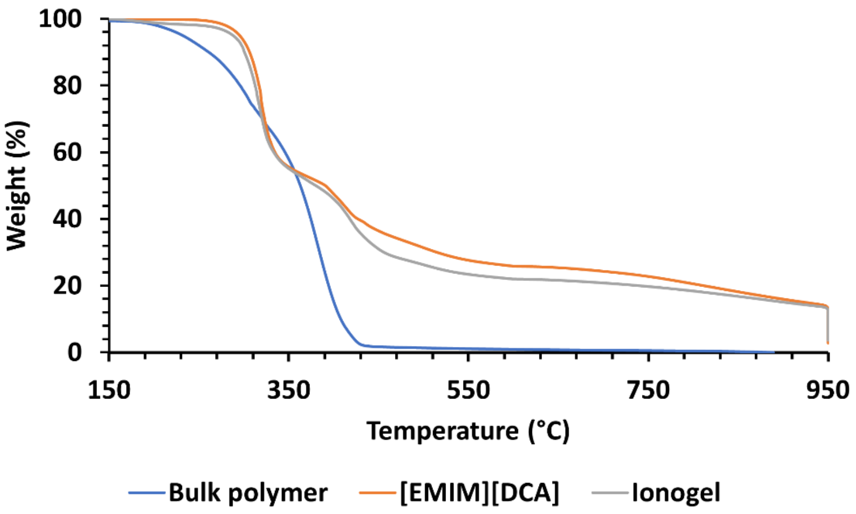

Figure S59. Thermogravimetric analysis (TGA) data obtained for bulk PHEMA$_{30}$-*b*-PBzMA$_{300}$ block copolymer (blue), pure [EMIM][DCA] (orange), and 15% w/w PHEMA$_{30}$-*b*-PBzMA$_{291}$ worm ionogel (grey).

References

1. J. Ilavsky and P. R. Jemian, *J. Appl. Crystallogr*, 2009, **42**, 347-353.
2. J. Pedersen, *J. Appl. Crystallogr*, 2000, **33**, 637-640.
3. J. S. Pedersen and M. C. Gerstenberg, *Colloids Surfaces A Physicochem. Eng. Asp.*, 2003, **213**, 175-187.
4. J. S. Pedersen, C. Svaneborg, K. Almdal, I. W. Hamley and R. N. Young, *Macromolecules*, 2003, **36**, 416-433.
5. L. J. Fetters, D. J. Lohsey and R. H. Colby, *Physical Properties of Polymers Handbook*, Springer, New York, 2nd edn., 2007.
6. J. S. Pedersen and P. Schurtenberger, *Macromolecules*, 1996, **29**, 7602-7612.
7. J. Bang, S. Jain, Z. Li, T. P. Lodge, J. S. Pedersen, E. Kesselman and Y. Talmon, *Macromolecules*, 2006, **39**, 1199-1208.
8. B. Hammouda, *Probing Nanoscale Structures – The SANS Toolbox*, 2008.
